# Rapid, low-input, low-bias construction of shotgun fragment libraries by high-density *in vitro *transposition

**DOI:** 10.1186/gb-2010-11-12-r119

**Published:** 2010-12-08

**Authors:** Andrew Adey, Hilary G Morrison, Asan, Xu Xun, Jacob O Kitzman, Emily H Turner, Bethany Stackhouse, Alexandra P MacKenzie, Nicholas C Caruccio, Xiuqing Zhang, Jay Shendure

**Affiliations:** 1 Department of Genome Sciences, University of Washington, Seattle, WA 98195, USA; 2 The Marine Biological Laboratory, Woods Hole, MA 02543, USA; 3 BGI-Shenzhen, Shenzhen 518000, China; 4 Epicentre Biotechnologies, 726 Post Road, Madison, WI 53713, USA

## Abstract

We characterize and extend a highly efficient method for constructing shotgun fragment libraries in which transposase catalyzes *in vitro *DNA fragmentation and adaptor incorporation simultaneously. We apply this method to sequencing a human genome and find that coverage biases are comparable to those of conventional protocols. We also extend its capabilities by developing protocols for sub-nanogram library construction, exome capture from 50 ng of input DNA, PCR-free and colony PCR library construction, and 96-plex sample indexing.

## Background

Massively parallel DNA sequencing methods are rapidly achieving broad adoption by the life sciences research community [1,2]. As the productivity of these platforms continues to grow with hardware and software optimizations, the bottleneck experienced by researchers is increasingly at the front end (the construction of sequencing libraries) and at the back end (data analysis and interpretation) rather than in the sequencing itself.

**Figure 1:**
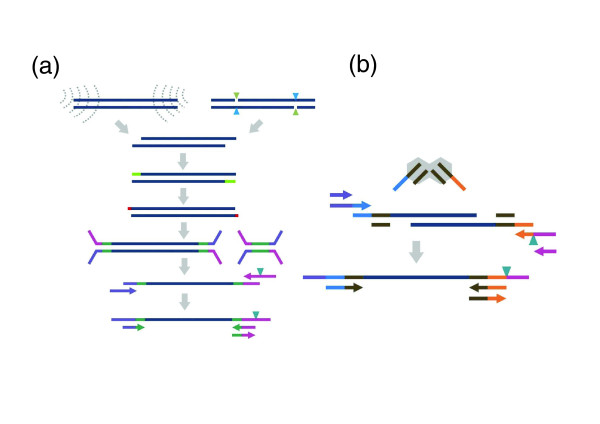
**Methods for constructing *in vitro *fragment libraries**. **(a) **In the conventional protocol, mechanical or endonuclease fragmentation is followed by end-polishing, A-tailing, adaptor ligation and PCR. **(b) **With transposase-mediated adaptor insertion, fragmentation and adaptor insertion occur in a single 5-min *in vitro *step, followed by PCR. For both methods, a primer-embedded sample-specific barcode can be incorporated during PCR amplification (black triangle). Dark blue: Genomic DNA. Light green: End repaired sequence. Red: A-tail. Magenta/dark green and purple/dark green: Adaptors. Mid blue/brown/orange: Transposase adaptors. Cyan/light green triangles: Endonuclease fragmentation. Grey curved dotted lines: Sonication. Grey hexagon: Transposase.

The input material for commonly used platforms, such as the Illumina Genome Analyzer [3], the Roche (454) Genome Sequencer [4], the Life Technologies SOLiD platform [5], as well as for 'real-time' third-generation sequencers such as Pacific Biosciences [6], consists of complex libraries of genome- or transcriptome-derived DNA fragments flanked by platform-specific adaptors. The standard method for constructing such libraries is entirely *in vitro *and typically includes fragmentation of DNA (mechanical or enzymatic), end-polishing, ligation of adaptor sequences, gel-based size-selection, and PCR amplification (Figure 1a). This core protocol may be preceded by additional steps depending on the specific application, such as cDNA synthesis for RNA-seq libraries [7].

Although generally effective, several aspects of the standard method are throughput-limiting or otherwise suboptimal. These include: (1) Labor: there are several labor-intensive enzymatic manipulations with obligate clean-up steps. (2) Time: the protocol requires 6-10 hours from beginning to end, often including an overnight incubation. (3) Automation: although 96-plex, semi-automated processing has been achieved by large-scale genome centers [8], many researchers lack access to the requisite robotic liquid handling systems and/or instruments for parallelized mechanical fragmentation. (4) Sample indexing: incorporation of barcoded adaptors, which enable concurrent analysis of multiple samples and post-sequencing deconvolution, still requires most steps to be carried out on individual samples prior to pooling [9]. (5) High input requirements: standard protocols for shotgun DNA sequencing suggest 1-10 μg DNA as input material per library. This is often not possible, for example in cancer genomics where sample material can be limited. (6) Coverage bias: biases in sequence coverage correlated with G+C content can arise from steps secondary to library construction, including gel purification [10] and PCR amplification [11]. Amplification-free versions of these protocols may reduce G+C biases and eliminate PCR duplicates [11,12], while potentially increasing input requirements.

In the alternative approach that we characterize and extend here, a hyperactive derivative of the Tn5 transposase is used to catalyze *in vitro *integration of synthetic oligonucleotides into target DNA at a high density ('Nextera', Epicentre, Madison, WI, USA). Wild-type Tn5 transposon DNA is flanked by two inverted IS50 elements, each containing two 19 bp sequences required for function (outside end and inside end). A 19 bp hyperactive derivative (mosaic end, ME) is sufficient for transposition provided that the intervening DNA is long enough to allow the two ends to come in close proximity in order to form a complex with a Tn5 transposase homodimer. The relatively low activity of the wild-type Tn5 transposase was cumulatively increased through several classes of mutation [13]. In a classical *in vitro *transposition reaction, hyperactive Tn5 transposomes (hyperactive transposase mutant bound to ME-flanked DNA) bind target DNA and catalyze the insertion of ME-flanked DNA into the target DNA with high frequency [14]. When free synthetic ME adaptors are used instead (isolated from one another, in contrast to ME-flanked DNA in which two ME sequences are linked by the intervening DNA), transposase activity results in fragmentation and end-joining of the synthetic ME adaptor to the 5' end of target DNA. To generate fragment libraries compatible with massively parallel DNA sequencing, limited-cycle PCR is used to append platform-specific primers (Figure 1b).

**Figure 2:**
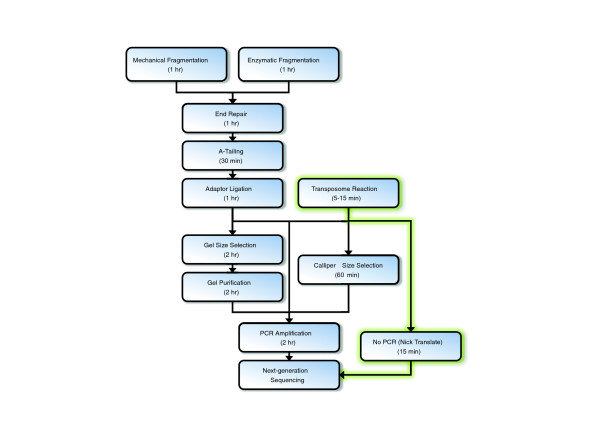
**Schematic of steps associated with different library preparation methods**. Transposase-catalyzed adaptor insertion significantly reduces the number of steps and time associated with library construction (green path).

Significant potential advantages of transposase-catalyzed adaptor insertion as a library preparation method, relative to conventional library preparation, include, firstly, many fewer steps, as the fragmentation, polishing, and ligation steps are replaced by a single 5-minute reaction and optional 10-minute pre-PCR clean-up (Figure 2). Libraries requiring particularly constrained insert size distributions (such as for *de novo *assembly) may optionally be subjected to chip- or gel-based size selection, increasing preparation time by 1 hour or 3-4 hours, respectively. The second advantage is greatly reduced input requirements while maintaining library complexity. This is expected to be possible because of a more efficient conversion of input DNA into sequencing-compatible material. However, these potential advantages are balanced by the competing concern that transposase-mediated fragmentation will introduce significant sequence-dependent biases relative to conventional library construction.

Here, we report the results of an extensive comparison of transposase-catalyzed fragmentation with standard library construction protocols. We also describe the development of several derivative protocols for transposase-catalyzed fragmentation that significantly extend its capabilities. To evaluate performance with respect to key parameters including sequence-dependent biases, we compared methods across several organisms and sequencing platforms, including whole genome sequencing of a cell line derived from a previously sequenced human, YH1 [15], on a single flow-cell with the Illumina HiSeq platform. New protocols reported here that extend the utility of this method include: (1) a 96-plex sample indexing scheme, validated on 96 bacterial genomes; (2) capture and sequencing of the complete coding exon content (exome) from 50 ng of input human genomic DNA; (3) a protocol for the construction and sequencing of shotgun libraries from as little as 10 pg of starting material; (4) a PCR-free version of the method that mitigates associated G+C biases and decreases the total time for library preparation time to less than 30 minutes; and (5) a method analogous to 'colony PCR' for single-step preparation of genomic sequencing libraries directly from bacterial colonies.

## Results

### Comparison of standard versus transposase-based protocols

We performed a side-by-side comparison of three protocols: (1) standard library construction with mechanical fragmentation; (2) standard library construction with time-dependent endonuclease-based fragmentation ('dsDNA fragmentase', NEB); and (3) transposase-catalyzed adaptor insertion ('Nextera', Epicentre). To evaluate performance on the Illumina platform, sequencing libraries and technical replicates were prepared from two genomic DNA samples (*Homo sapiens *NA18507, *Escherichia coli *CC118) with each of the three methods. Paired-end, 36 bp reads were generated on an Illumina Genome Analyzer IIx (GAIIx). Reads were mapped using BWA [16] to the *E. coli *genome (K12) or human genome (hg18) as appropriate. To evaluate performance on the Roche (454) platform, sequencing libraries were constructed from two bacteriophage DNAs (CRW10 and PA1) with each of the three methods. Libraries were sequenced on a Roche (454) Genome Sequencer FLX, followed by *de novo *assembly (gsAssembler) and read mapping (gsMapper) to the appropriate reference genome. A summary of samples processed and sequence data generated on both platforms is provided in Table S1 in Additional file 1.

Sites of mechanical fragmentation, endonuclease fragmentation, and transposase-catalyzed adaptor insertion were characterized by calculating nucleotide composition in the vicinity of the mapping position of the first base of each sequence read (the fragmentation site; Figure S1 in Additional file 2). This revealed a slight but highly correlated bias for mechanical and endonuclease fragmentation, which suggests that most bias for these two methods is introduced after these protocols converge (for example with A-tailing or adaptor ligation), and that both mechanical fragmentation (here, either acoustic sonication or nebulization) and endonuclease fragmentation (with dsDNA fragmentase) have very low intrinsic biases. In contrast, a more extended signature is observed for sites of transposase-catalyzed adaptor insertion, weakly resembling the reported insertion preference of the native Tn5 transposase (AGNTYWRANCT, where N is any nucleotide, R is A or G, W is A or T, and Y is C or T) [17]. However, when calculated in terms of per-position information content, the bias of transposase-catalyzed adaptor insertion is low, and only slightly greater than the other protocols. For *E. coli *data, maxima of per-position information content over ± 10 bp, on a two-bit scale for fixed positions, are 0.10, 0.11, and 0.16 for mechanical fragmentation, endonuclease fragmentation, and transposase-catalyzed adaptor insertion, respectively. Average information content over ± 10 bp are 0.0056, 0.018, and 0.049, respectively. Equivalently low information contents were observed for human and phage libraries (Table S2 in Additional file 1). The effective bias associated with transposase-catalyzed adaptor insertion is thus greater than with standard library construction, but only modestly so. For *E. coli *and human libraries, signatures of bias were consistent in technical replicates for all three methods.

**Figure 3:**
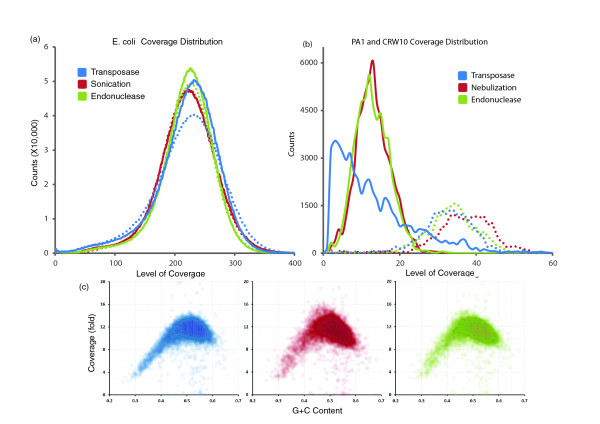
**Comparison of coverage bias**. **(a) **Coverage distribution across the *E. coli *genome with transposase (blue), sonication (red), and endonuclease (green) methods (solid lines) and replicates (dotted lines), normalized for total sequencing depth. **(b) **Coverage distribution across the PA1 and CRW10 bacteriophage genomes with transposase (blue), nebulization (red), and endonuclease (green) methods (dotted lines represent replicate libraries). **(c) **G+C bias for *E. coli *was assessed by calculating G+C content of the reference in 500 bp bins and plotting the coverage in each for transposase (blue), sonication (red), and endonuclease (green) methods, all of which show an approximately equivalent bias against the extremes.

The greater insertion bias is problematic in a practical sense only if it has a significant impact on the distribution of genomic coverage. Consistent with the low calculated information content of the observed biases, the gross distributions of genomic coverage observed for the three methods are very similar (Figure 3a, b), the exception being the PA1 bacteriophage library, which may be skewed as a result of sequence context in a relatively small genome. Furthermore, similar biases in coverage are observed for different G+C content bins, with reduced representation at both extremes (Figure 3c). As PCR was used to prepare libraries constructed with all three methods, the consistent G+C bias probably arises at that step [11]. We initially predicted that the similar genomic coverage distribution associated with each method was due to factors introduced after the three protocols converge on common steps (solution phase PCR, cluster PCR, and sequencing). However, the correlation in coverage between methods at a per-base level was modest, with transposase-catalyzed adaptor insertion the least correlated with the other methods (Table S3 in Additional file 1).

**Figure 4:**
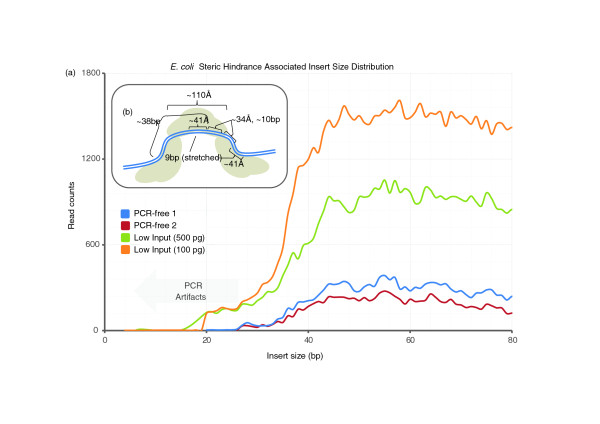
**Insert size showing steric hindrance**. **(a) **Insert size was generated from libraries spiked into a paired-end 101 bp run resulting in a large proportion of reads reading into the adaptor sequence. Tails of reads were then aligned to one another to discern the insert size between adaptors, resulting in a mapping-independent insert size at the lower extreme. All reads with an insert size less than 25 bp were PCR artifacts. **(b) **The noticeable drop below 40 bp is consistent with a model for complete saturation of transposition events on a given stretch of DNA. The roughly 110 Å transposase homodimer (grey) is bound to genomic DNA (blue), such that the core of the enzyme acts on a 9 bp region drawn out to 41 Å as well as approximately 10 additional bases of DNA flanking either side (~34 Å each) that are essentially protected from a subsequent transposase attack due to steric hindrance. Since the core region is duplicated in the process, the minimum spacing of transposition events is approximately 38 bp.

In this comparative analysis, libraries generated by transposase-catalyzed adaptor insertion were sequenced directly after PCR (without size-selection), and the observed insert size distribution was considerably shorter than the other, size-selected, methods (transposase: 100 ± 47 bp, sonication: 256 ± 48 bp, endonuclease: 244 ± 56 bp; Figure S2 in Additional file 2). To evaluate whether a lower-bound on insert size exists, tails of long-read (101 bp) pairs were aligned to one another and a mapping-independent size distribution constructed, revealing a sharp decrease at about 35 bp that is probably a secondary consequence of steric hindrance of adjacent, attacking transposases (Figure 4). This phenomenon also explains the about 10 bp peaks at the lower end of the insert size distribution resulting from the helical pitch of the DNA as it extends away from the transposase.

With alternative buffer and reaction conditions, other target size ranges can be achieved. For example, the transposon method adapted for Roche (454) library construction resulted in significantly longer fragments (300-800 bp; Figure S3 in Additional file 2). To assess whether fragment size of libraries generated by transposase-catalyzed adaptor insertion could be constrained without resorting to gel-based size-selection, we evaluated alternative buffer and reaction conditions in combination with different approaches to post-PCR sample clean-up (Figure S4 in Additional file 2). Notably, an automated chip-based size-selection yielded well-constrained libraries (insert size 162 ± 28 bp).

### Whole genome sequencing of human and *Drosophila *genomes

To assess performance further, we conducted whole genome sequencing on transposase-based libraries from *H. sapiens *and *Drosophila melanogaster*. Human genomic DNA from a previously sequenced individual, YH1 [15], was used to generate a series of libraries under different reaction conditions and size-selections that were then subjected to seven lanes of paired-end 90 bp (PE90) sequencing on the Illumina HiSeq platform. Of 934 million reads, 781 million were mapped [16] to the human genome (hg18) for 25× coverage. Although a total of seven libraries were constructed and sequenced to assess reproducibility, the complexity of each individual library was sufficient enough that whole genome sequencing could be carried out using a single library. Variant calling on mapped YH1 data was performed with *samtools *[18] requiring consensus Q30 at called positions (Figure S5 in Additional file 2). By these criteria, 3,556,679 SNPs were called (87% in dbSNP129; transition/transversion ratio (Ti/Tv) = 2.07), substantially greater than the 3,074,097 SNPs reported in initial sequencing of YH1. There were 2,922,525 SNPs shared between the analyses (91% in dbSNP129; Ti/Tv = 2.07), 634,154 SNPs unique to our analysis of this genome (70% in dbSNP129; Ti/Tv = 2.08), and 151,572 SNPs unique to the initial analysis of this genome (65% in dbSNP129; Ti/Tv = 1.18). The larger number of SNPs identified here may follow in part from greater mappability with longer read-lengths.

In this analysis, cell-line DNA derived from lymphoblasts was used; however, original sequencing of YH1 by Wang *et al. *(2008) [15] was carried out on blood DNA. Notably, 4,036 positions were called as mutations in the cell line and as the reference base in blood, both at a high quality score (30) and in uniquely mappable regions of the genome (see Methods). Of the 1,720 SNPs at a quality over 50 (Ti/Tv = 0.95), a randomly selected 100 were subjected to validation in DNA from blood, DNA from the primary culture used to generate the cell line, and DNA from the cell line. Interestingly, 63 were confirmed as mutations only in the cell line (Ti/Tv = 1.1; one failed assay in primary culture). Of the 37 positions that failed validation, 31 were confirmed as the reference base in blood, primary culture, and cell line (Ti/Tv = 0.48), and the remaining six positions were variant in all three (Ti/Tv = 1.0; one failed assay in primary cell culture). Further experimentation is required to determine whether the validated mutations observed in the cell line represent only mutations occurring during immortalization or propagation of the cell line, or the eventual fixation of somatic mutations present at very low frequencies in primary culture.

**Figure 5:**
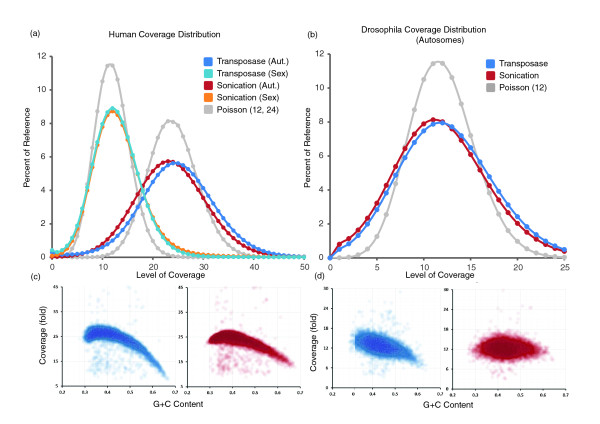
**Sequence coverage of human and *Drosophila***. **(a) **Coverage distribution as a percentage of the genome for human (YH1) using transposase (dark blue, autosomes; light blue, sex chromosomes) and sonication [15] (down-sampled to equivalent coverage; red, autosomes; orange, sex chromosomes) methods. Poisson (no bias) distributions (gray) with λ = 12 (sex chromosomes) and λ = 24 (autosomes) are also shown. Poisson distribution is the expected if there were absolutely no bias. **(b) **Coverage distribution as a percentage of *Drosophila *autosomes using transposase (blue, down-sampled to equivalent coverage) and sonication (*Drosophila *Population Genomics Project (DPGP), red) methods, as well as Poisson distribution with λ = 12 (gray). **(c,d) **Coverage with respect to G+C content of the reference in 10 kb or 1 kb bins for (c) human (YH1) and (d) *Drosophila *genomes respectively, for transposase (blue) and sonication (red) methods at comparable global genomic coverage.

Importantly, coverage of YH1's genome in sequencing of libraries derived from transposase-catalyzed fragmentation was relatively uniform when compared with the data generated on this same individual from conventional libraries (Figure 5a). The observed GC bias in whole human genome sequencing data from the two methods was comparable (Figure 5c); however, a modest decrease (23%) in coverage at bins with high GC content (≥60%) was observed with the transposase method. This decrease can potentially be mitigated by a PCR-free version of this method (discussed below), or by alternative PCR conditions (data not shown). For the *Drosophila *genome (dm3), one lane of PE45 sequencing of a transposase-based library on an Illumina GAIIx yielded 16× mean coverage. As with the human genome, the distribution of coverage was largely equivalent to that observed in sequencing *Drosophila *with standard libraries (Figure 5b, d) along with the modest decrease in coverage for regions of high GC content. For both human and *Drosophila *genomes, the signature of bias in the vicinity of insertion sites was similar to that observed in the comparative analysis.

**Figure 6:**
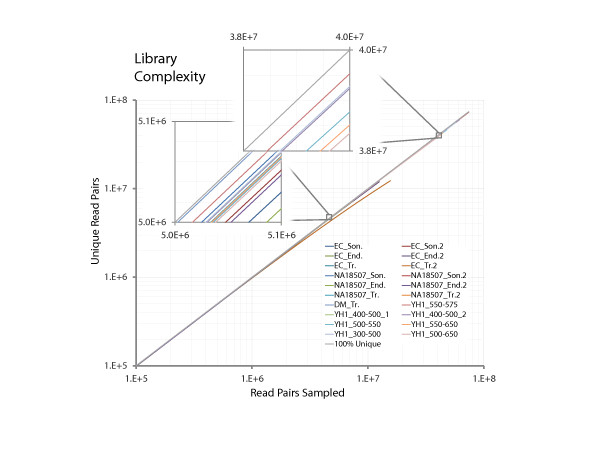
**Library complexity**. Library complexity for each library shown by incremental, random sampling of 50,000 reads, without replacement, and plotting (on log-log scale) the number of uniquely occurring read-pairs with respect to total number of sampled read-pairs. Species: DM, *Drosophila*; EC, *E. coli*; NA18507 and YH1, human. Methods for fragmentation: End., endonuclease; Son., sonication; Tr., transposase. Size selection ranges are given for the YH1 libraries (all these were generated using transposase. Libararies ending in "2" are replicate libraries. 100% uniqueis in gray, i.e. the distribution if there were no duplicates of any sort.

Complexity, that is, the number of molecules of distinct origin, is a critical aspect of shotgun library quality, especially for libraries that will be deeply sampled or subjected to further bottlenecking, such as hybrid capture or size selection. Low complexity manifests as an excess of duplicate reads with identical mapping coordinates, which arise from the same progenitor molecule and can thus skew downstream analyses including SNP calling and genotyping. The complexity of each prepared library here was analyzed by incremental sampling of 50,000 read-pairs without replacement and plotting the number of read-pairs sampled versus the number of unique read-pairs (as determined by mapping location) within the sample. In this analysis, the extent of deviation from linearity provides a measurement of sample complexity. All human genome and *Drosophila *genome libraries sequenced here were found to be highly complex, each comprising over 96% unique read-pairs, even as sequencing depths approached 100 million read-pairs, indicating a single such library could be used for whole genome sequencing (Figure 6). The high complexities achieved are consistent with a high efficiency of conversion of input mass into sequence-compatible material as compared with conventional library construction. Less complexity was consistently observed for all *E. coli *libraries, but this is likely to be because we are simply saturating the set of possibilities for start-point pairs that are used to identify PCR duplicates by deep sequencing of a small genome.

### Low input targeted sequence capture of the human exome

Exome capture is an increasingly mature technology, but standard protocols require several micrograms of input genomic DNA, which can be problematic when sample is limiting (for example with tumor samples). We subjected a library from 50 ng human genomic DNA by transposome fragmentation to exome capture (Nimblegen SeqCap EZ Exome probes v1.0). Because the adaptor sequences are different from those in libraries prepared using mechanical shearing, custom blocking oligonucleotides were designed and used. After capture, the library was subjected to pre-sequencing real-time PCR with standard primers followed by sequencing on an Illumina GAIIx (SE36). The resulting reads were aligned to the human genome (hg18) with 78% mapping, of which 47% fell within 100 bp of a targeted exon (Figure S6 in Additional file 2). A direct comparison with an equivalent number of mapped SE36 reads from a standard library, after capture with the same kit, revealed nearly identical complexity for on-target reads (41% and 43% of an equivalent number of on-target SE36 reads with unique start sites for transposome-based and standard libraries, respectively), as well as comparable uniformity (87% and 82% of target bases covered with ≥1 reads for transposome-based and standard libraries, respectively). However, specificity was notably lower (47% of reads on or near target for transposome-based libraries versus 80% for standard libraries). Nonetheless, we note that the standard protocol has been extensively optimized in the context of production-level scaling, and it is likely that specificity in the capture of transposome-based libraries can also be improved upon. Furthermore, the disadvantage of lower specificity is balanced by the advantage of significantly lower input requirements for genomic DNA entering a targeted capture workflow (50 ng for transposase-based libraries versus 3 μg for standard libraries).

### Sub-nanogram library construction

To push the limits on library construction using reduced starting material, *E. coli *libraries were generated from 500 pg and 100 pg genomic DNA and sequenced as part of a barcode pool. For each library, expected numbers of read counts were observed (0.5 and 0.6 million mapped reads, respectively) without a noticeable drop in complexity (both libraries over 98% at 0.5 million read-pairs), or coverage uniformity. Next, we generated a library from 10 pg human genomic DNA, or roughly three copies of the human genome, which produced over 2 million uniquely mapped read-pairs. Although complexity was reduced because of the significant decrease in progenitor molecules entering PCR, the potential advantages of sequencing from material approaching a single equivalent of the human genome are substantial.

### PCR-free library construction

**Figure 7:**
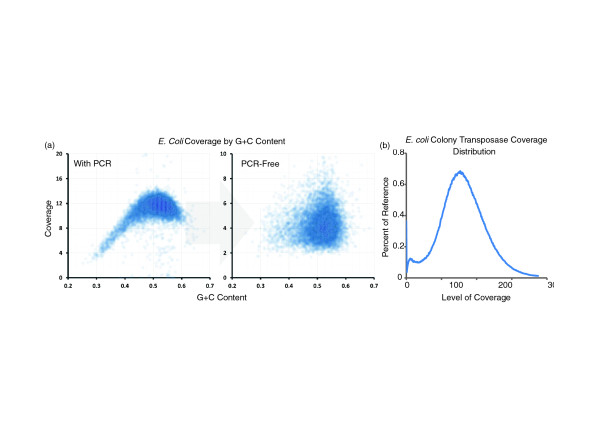
**PCR-free reduction in G+C coverage bias and direct-from-colony coverage distribution**. **(a) **Coverage with respect to G+C content in *E. coli *with and without PCR was assessed by calculating G+C content of the reference in 500 bp bins and plotting the coverage in each for transposase after PCR (left) and with the PCR-free method (right). A significant reduction in coverage bias at the extremes of G+C content is observed. **(b) **Coverage distribution for *E. coli *library prepared directly from cell lysate without purification.

Standard sequencing libraries for the Illumina platform have been generated without the use of PCR amplification in order to reduce associated biases [11,12]. We developed a similar approach for transposase-based methods by including sequences corresponding to the primers used for cluster formation, i.e. the Illumina adaptor sequences, into the adaptors that are added during the transposition reaction, as opposed to incorporating them during PCR (See Methods). After transposition, a nick translation is performed resulting in Illumina-ready libraries. This method was used to sequence *E. coli *CC118 and human NA18507 with two replicates of each using 100 ng and 200 ng starting material. A noticeable decrease in G+C coverage bias was observed (Figure 7a). Furthermore, complexity for each of these libraries was over 98%. The development of PCR-free, transposase-based library construction reduces the full amount of time required for converting DNA to a sequencing-ready shotgun library to less than 30 minutes.

### 96-plex sample indexing

Second-generation sequencing platforms suffer from poor granularity. For example, in the data described above, a single PE90 lane on the Illumina HiSeq platform yielded 20 Gb of mappable sequence, which is far in excess of what is required for many projects. Sample indexing (or 'barcoding') is a useful solution, but reported protocols still require most steps of library preparation to be carried out on individual samples prior to pooling, and also can suffer from non-uniform performance of individual barcodes [9]. With transposase-catalyzed adaptor insertion, sample indexing could potentially be introduced during adaptor insertion, or during the subsequent PCR step, that is, using a primer-embedded barcode sequence (Figure 1b). To evaluate the compatibility of this method with indexing, we attempted the latter approach. Ninety-six barcodes (9 bp) were designed with a minimal edit distance of four between all pairs and additional constraints on base composition (Table S4 in Additional file 3). Performance was evaluated by subjecting DNA from 96 evolved derivatives of *Pseudomonas aeruginosa *to independent library construction, each with a different barcode-embedded primer during PCR. Post-PCR amplicons were quantified and pooled, followed by several lanes of massively parallel sequencing (PE76, with a third read to collect the 9 bp index). Samples were deconvolved using 9 bp indexes: 92%, 3%, and 3% reads were assigned with 0, 1, and 2 mismatches, respectively, and only 4% could not be unambiguously assigned. With the exception of a few outliers, the distribution of barcode assignments across the 96 was relatively uniform with 90% within a fourfold range (Figure S7 in Additional file 2), as was the proportion of reads mapping to the reference, illustrating the robustness of the library protocol and the indexing scheme.

### Constructing genomic libraries directly from bacterial colonies

In evaluating 96-plex sample indexing for 96-plex bacterial genome sequencing with transposase-catalyzed adaptor insertion, the burden of technical effort shifted from library preparation to the isolation of genomic DNA from each isolate. We speculated that integration of the transposase reaction into a 'colony PCR'-like workflow (in which cells from bacterial colonies are directly mixed into a PCR reaction without DNA isolation) could be used to further simplify library preparation for bacterial genome sequencing (Figure S8 in Additional file 2). A pipet tip was used to transfer a small number of cells directly from an *E. coli *colony transformed with pUC19 to a transposase fragmentation reaction (with heat-lysing prior to addition of enzyme). An aliquot of this reaction served as input for PCR amplification without any intervening clean-up step. Sequencing (SE36) yielded 27 million reads, for 170× coverage of the *E. coli *genome (81% of reads) and 37,000× coverage of pUC19 (10% of reads). The remaining 8% mapped to the F plasmid, or an insert cloned into pUC19, or remained unmapped. Coverage of the *E. coli *genome was uniform (Figure 7b). We propose that direct preparation of genomic sequencing libraries from bacterial colonies with no DNA isolation or intervening purification steps will be useful for rapidly preparing sequencing-ready, indexed fragment libraries from large numbers of bacterial isolates.

## Discussion

Massively parallel sequencing platforms generally require the conversion of genomic DNA (or other nucleic acid sample) into a fragment library that includes common adaptor sequences that are used to mediate clonal amplification and/or the priming of sequencing reactions. Practical limitations of conventional approaches to generating these libraries include high requirements for labor, time and cost, as well as the low efficiency of mass conversion into sequencing-compatible material. Here, we evaluate an alternative approach in which transposase catalyzes the fragmentation of target DNA and insertion of adaptor sequences in a 5-minute, small-volume reaction. The workflow is thus markedly simpler than the conventional approach yielding significant savings in terms of time and labor (summarized in Figure 2). The input requirements are also over an order of magnitude lower than what is typically used with standard methods, and we demonstrate that high complexity libraries can be generated from as little as 100 pg of input DNA. Furthermore, input can be reduced to as low as just a few copies of the human genome and still produce a significant amount of sequence data. Taking advantage of the simplicity and low input requirements, we developed a method to construct libraries directly from bacterial colonies without DNA isolation or intervening clean-up steps.

Although there are significant advantages, this method nonetheless has its limitations. First, although there is a significant reduction in required steps and time, preparing very large numbers of libraries is still challenging without some degree of automation. Second, one has relatively limited control over the size distribution of fragmentation. In general, the trend is that the insert size distribution is smaller than desired when reacting to completion, and broader than desired when altering reaction conditions to increase the mean insert size. Third, genomes with high G+C contents show greater bias with this method than with conventional methods, although this is potentially correctable in part by the PCR-free approach or through modified PCR conditions. A related point is that we have also observed that performing transposase-catalyzed adaptor insertion on a single PCR product results in significantly greater bias than with shotgun libraries (J. Hiatt, personal communication), potentially secondary to a high molar concentration of a limited number of possible insertion sites. Fourth, we note that this library preparation does not solve an ongoing challenge in the field, which is how best to efficiently construct high-complexity, long-insert mate-paired libraries.

A comparison of sequence composition in the vicinity of fragmentation sites identified a bias signature that was larger and more extended than that associated with conventional methods. However, we found that this had little impact at the level of coverage for bacterial, human, and *Drosophila *genomes. Nonetheless, the observed impact may be greater in genomes with compositions that correlate with this bias pattern, or in smaller genomes such as bacteriophage. In our view, for most goals, the disadvantage of the slightly greater bias is offset by the large advantages that we observed with respect to speed, simplicity, and low input requirements.

A further reduction in preparation time was achieved in developing a PCR-free, transposase-based library construction method. This approach also decreased biases resulting from amplification, notably in coverage with respect to G+C content. Looking ahead, we anticipate that the partnership of extremely fast, simplified methods for library construction with third-generation 'real-time' sequencing methods may represent a critical path forward in reducing the time between acquiring biological material and obtaining analyzed sequence data to less than 1 hour.

## Conclusions

Current methods for preparing *in vitro *DNA fragment libraries for massively parallel sequencing are suboptimal for projects involving limited amounts of starting material or large numbers of samples. Here we have characterized an alternative method of library construction in which highly active transposase catalyzes the simultaneous fragmentation and adaptor ligation in a single 5-minute incubation. Comparison with conventional methods of library preparation, relying on mechanical or endonuclease fragmentation, finds that although transposase-catalyzed adaptor insertion demonstrates a slightly greater insertion bias, this has little impact at the level of genomic coverage and is offset by large advantages with respect to speed, simplicity, and low input requirements.

To fully take advantage of the method and expand on transposase-catalyzed adaptor insertion we applied it on a larger scale, including sequencing a human genome on a single flow-cell of a massively parallel sequencing platform, and validating a 96-plex sample indexing scheme. We also show that transposase-catalyzed adaptor insertion can be integrated with exome sequencing workflows, with the advantage of significantly lower input requirements relative to conventional protocols for targeted sequence capture (50 ng versus several micrograms). In addition, we have generated libraries from as little as 10 pg of starting material and also developed a PCR-free library construction method in order to reduce associated biases and further reduce preparation time to less than 30 minutes. Finally, we demonstrate the direct preparation of genomic sequencing libraries from bacterial colonies with no DNA isolation or intervening purification steps.

## Materials and methods

### Genomic DNA

Genomic DNA for *H. sapiens *NA18507 (Yoruban) was prepared by the Coriell Institute. *E. coli *CC118 (MC1000 (*araD139 *Δ(*ara leu*)*7697 *Δ*lacX74 phoAΔ20 galE galK thi rpsE rpoB argE_am _recA1*)) [19], genomic DNA, and CRW10 and PA1 phage genomic DNA were extracted using Qiagen (Valencia, CA, USA) MidiPrep buffers P1, P2, P3 and were cleaned by phenol, purified from low melting point agarose and dissolved in Tris/EDTA (TE) buffer. YH1 genomic DNA was extracted from a lymphoblastoid cell line of Yanhuang [15] using protein K and phenol/chloroform [20] and further subjected to RNase treatment/purification. The *D. melanogaster *genomic DNA was extracted from whole bodies of several individuals by Puregene blood core kit B (Qiagen). The molecular weights of both *Drosophila *and YH1 genomic DNAs were confirmed to be larger than 23 kb by gel electrophoresis (not shown), with no detection of degradation or RNA/protein contamination, and quantified by Quant-iT dsDNA HS assay kit (0.2-100 ng; Invitrogen, Q32854, Carlsbad, CA, USA); of each, an aliquot was diluted to 25 ng/μl for use in library construction. *P. aeruginosa *PAO1 strains were selected for tobramycin resistance at 16 mg/l (41 strains), ciprofloxacin resistance at 4 mg/l (47 strains), or no antibiotic resistance (8 strains). DNA was then isolated using the Wizard^® ^SV 96 genomic DNA purification system (Promega, Madison, WI, USA). Concentrations of isolated DNA were measured using a Nanodrop (Thermo Scientific, Waltham, MA, USA).

### Mechanical shearing

Shearing was performed on two 5-μg aliquots of *E. coli *CC118 genomic DNA brought up to 200 μl with TE. Sonication was performed by two 15-min treatments with a Bioruptor sonicator (Wolf Laboratories, Pocklington, UK) at maximum settings in a cold room, switching out the water between treatments to keep samples cool. After sonication, each sample was cleaned up using a QIAquick PCR purification kit, eluting in 30 μl Buffer EB (Qiagen). CRW10 and PA1 phage DNA was fragmented using 5 μg aliquots brought to 100 μl with TE and mixed with 500 μl nebulization buffer (Roche/454, Branford, CT, USA) in a nebulizer cup. Nebulization was carried out using 45 psi (310 kPa) nitrogen for 1 min on ice. The sheared DNA was cleaned and concentrated using MinElute columns and eluting in Buffer EB (Qiagen).

### Enzymatic fragmentation

Enzymatic fragmentation was performed on six 1 μg aliquots of genomic DNA from both *E. coli *CC118 and *H. sapiens *NA18507 added to 2 μl 10× fragmentation reaction buffer, 0.2 μl 100× BSA (NEBiolabs, Ipswich, MA, USA), 2 μl NEB fragmentase enzyme, and nuclease-free water (Ambion, Austin, TX, USA) to 20 μl. Reactions were gently vortexed and spun-down, then incubated on ice for 5 min followed by a time-course incubation at 37°C, removing samples at 15, 20, 30, 45, 60, and 120 min for each of the two sets. Reactions were stopped by placing on ice followed by purification using a QIAquick PCR purification kit, eluting in 30 μl buffer EB (Qiagen). 4 μl of each sample was run on a Novex TBE gel (Invitrogen) to observe size distribution. The 60-min time point showed the desired size range and duplicate samples were prepared by the same method for each organism. Fragmentation of phage DNAs were performed using 1 μg aliquots of PA1 and CRW10 in a 20 μl reaction volume including fragmentation reaction buffer to 1×, BSA, and 2 μl NEB fragmentase enzyme (NEB). The reaction was incubated on ice for 5 min followed by incubation at 37°C for 20 min and stopped with 5 μl 500 mM EDTA and cleaned using MinElute columns (Qiagen).

### Post-fragmentation library preparation

Post-fragmentation library preparation on the duplicate samples of *E. coli *CC118 sonication and fragmentase (60 min), and *H. sapiens *NA18507 fragmentase (60 min), was carried out as per standard Illumina methods, including a size selection using Novex TBE gels (Invitrogen), excising the 400-500 bp band. Final PCR amplification was carried out on a Bio-Rad (Hercules, CA, USA) MiniOpticon using SYBR Green I as a dye to monitor amplification. 1 μl of each final library was run on a Novex TBE gel (Invitrogen) for library size confirmation. Nebulized or fragmentase-treated phage samples were size-selected using SPRI beads (Beckman Coulter, Danvers, MA, USA) and used to construct libraries according to standard protocols, including end-polishing, adaptor ligation, fill-in, and single-strand isolation. Adaptor sequences included multiplex identifiers (barcodes).

### Transposase-based library preparation

Transposase-based library preparation for *E. coli *CC118 and *H. sapiens *NA18507 Illumina-compatible libraries used 50 ng of genomic DNA brought up to 15 μl in nuclease-free water (Ambion) followed by the addition of 4 μl 5× LMW Nextera reaction buffer and 1 μl Nextera enzyme mix (Illumina-compatible; Epicentre), followed by a gentle vortex and brief centrifugation. Each reaction tube was incubated at 55°C in a thermocycler with a heated lid for 5 min followed by placement on ice and immediate purification using a QIAquick PCR purification kit and elution in 20 μl buffer EB (Qiagen). Suppression PCR was then carried out using 10 μl of the eluate as template with 11.5 μl nuclease-free water (Ambion), 25 μl 2× Nextera PCR buffer, 0.5 μl SYBR Green, 1 μl 50× Nextera primer cocktail (Illumina-compatible), 1 μl 50× Nextera adaptor 2 (barcodes 1-2 for *E. coli *and 3-4 for *H. sapiens*), and 1 μl Nextera PCR enzyme. The reaction was cycled in a Bio-Rad MiniOpticon to monitor the reaction under the following conditions: (1×) 3:00 min at 72°C and (1×) 0:30 min at 95°C, followed by 13 cycles of [0:10 min at 95°C, 0:30 min at 62°C, 3:00 min at 72°C] for *E. coli *barcodes 1 and 2 and *H. sapiens *barcodes 3 and 4 (12 cycles for *H. sapiens *barcode 4). 1 μl of each post-PCR library was electrophoresed through a Novex TBE gel (Invitrogen) for library size confirmation. Size selection of the *E. coli *CC118 transposase libraries was carried out at Epicentre Biotechnology using Agencourt AMPure (> 300 bp size selection), Zymo DNA purification (no size selection), or Caliper (350 ± 10% bp size selection) methods. The *D. melanogaster *library was constructed by pooling two standard Nextera reactions following the manufacturer's protocol (Epicentre). For each reaction, 50 ng genomic DNA was initially tagmented (*in vitro *transposase-catalyzed adaptor insertion) at 55°C for 5 min, and then MinElute purified. This was followed by PCR amplification with same conditions as with *H. sapiens *and *E. coli *libraries for 12 cycles. 400-450 bp gel-based size selection was carried out prior to sequencing.

A total of seven *H. sapiens *YH1 libraries were constructed, differing in mass of DNA, number of PCR cycles, and selected DNA fragment size. These included two (about 500 bp and about 550 bp) produced from pooling five standard Nextera reactions, three (400-500 bp, 500-550 bp and 550-600 bp) produced from pooling two modified reactions with nine cycles of PCR enrichment, and another two libraries (300-500 bp and 500-650 bp) from a single tagmentation reaction using 500 ng starting DNA with five cycles of PCR enrichment. The insert-size distribution and final yields for the *Drosophila *and *H. sapiens *YH1 libraries were validated separately using a 2100 Bioanalyzer (DNA 1000 and 7500 kit; Agilent, Santa Clara, CA, USA) and quantitative PCR.

*P. aeruginosa *PAO1 Illumina-compatible shotgun libraries were prepared for each strain using Epicentre Biotechnologies' Nextera DNA sample preparation kits with a customized, unique 9 bp barcode sequence for each strain. The tagmentation reaction consisted of 200 ng PAO1 DNA, 25 μl Nextera high molecular weight buffer, 1 μl Nextera transposase enzyme, and water to a total volume of 20 μl. The reaction was incubated for 5 min at 55°C, cleaned using Qiagen MiniElute columns, and eluted in 11 μl water. PCR reactions included 5 μl of the fragmented DNA, 17 μl water, 25 μl Nextera PCR buffer, 1 μl Nextera PCR enzyme, 1 μl of a Nextera primer cocktail containing two short primers (at 10 μM each) and one long Illumina-compatible adaptor (at 5 μM), and 1 μl of the barcode containing Illumina adaptor (at 5 μM). PCR conditions used were the same as above using 12 cycles of amplification, followed by MinElute clean-up as before. Samples were run on a Novex TBE polyacrylamide gel to confirm library quality, and DNA concentrations measured using a Nanodrop.

For the Roche (454)-compatible libraries, standard Nextera reaction conditions were used with 50 ng CRW10 (barcode 11) or PA1 (barcode 10) bacteriophage DNA, 454-Titanium compatible kit components and standard PCR methods, cycling 15 times. The PCR products were purified using Qiagen MinElute columns. Library fragment sizes were assessed using an Agilent Bioanalyzer DNA1000 chip.

### Targeted sequence capture of the human exome

Libraries were prepared by transposase-catalyzed adaptor insertion by previously described methods using 50 ng genomic DNA (BK229.03, SFARI-SSC), 1 μl transposomes, 4 μl 5× HMW buffer, and water to 20 μl. Samples were incubated at 55°C for 5 min then cleaned up (AMPure) and eluted in 20 μl followed by the addition of 25 μl 2× Nextera PCR buffer, 1 μl 50× Nextera primer cocktail, 1 μl Nextera PCR enzyme, 0.5 μl 100× SYBR Green, and 1 μl of a barcoded adaptor (Table S4 in Additional file 3) with water to 50 μl. Reactions were carried out on a Bio-Rad MiniOpticon using recommended cycling conditions for 12 rounds. Each tube was cleaned up (AMPure, Agencourt, Boston, MA, USA) and checked for size and quantity on an Agilent Bioanalyzer DNA 1000 chip. One sample was selected for capture using all of the 414.6 ng for hybridization to Nimblegen (Madison, WI, USA) SeqCap EZ Exome probes v1.0 as per Nimblegen protocols using custom blockers (Nextera_Block1: 5'-AAT GAT ACG GCG ACC ACC GAG ATC TAC ACG CCT CCC TCG CGC CAT CAG AGA TGT GTA TAA GAG ACA G-3', Nextera_Block1_REV: 5'-CTG TCT CTT ATA CAC ATC TCT GAT GGC GCG AGG GAG GCG TGT AGA TCT CGG TGG TCG CCG TAT CAT T-3', Nextera_Block2: 5'-CAA GCA GAA GAC GGC ATA CGA GAT CGG TCT GCC TTG CCA GCC CGC TCA GAG ATG TGT ATA AGA GAC AG-3', Nextera_Block2_REV: 5'-CTG TCT CTT ATA CAC ATC TCT GAG CGG GCT GGC AAG GCA GAC CGA TCT CGT ATG CCG TCT TCT GCT TG-3') for 72 h at 47°C. After hybridization, wash was performed as per Nimblegen protocols with streptavidin-coupled magnetic beads. Finally, PCR amplification was performed on exome captured library (Post_Cap_Short_For_Amp: 5'-AAT GAT ACG GCG ACC ACC GAG ATC T-3', Post_Cap_Short_Rev_Amp: 5'-CAA GCA GAA GAC GGC ATA CGA GAT-3'; 1× [0:30 min at 98°C], 17× [0:10 min at 98°C, 0:30 min at 65°C, 0:45 min at 72°C]) followed by clean up (AMPure) and sequencing on an Illumina GAIIx SE36 run.

### PCR-free library preparation

Adaptor sequences (NoPCR1: 5'-AAT GAT ACG GCG ACC ACC GAG ATC TAC ACG CCT CCC TCG CGC CAT CAG AGA TGT GTA TAA GAG ACA G-3', and NoPCR2: 5'-CAA GCA GAA GAC GGC ATA CGA GAT CGG TCT GCC TTG CCA GCC CGC TCA GAG ATG TGT ATA AGA GAC AG-3') were designed to contain the original 'Nextera' adaptor sequences, but with an additional 5' overhang of either P1 or P2 on adaptor 1 or adaptor 2, respectively (i.e. sequences to make compatible with cluster PCR on Illumina flow-cell), thus eliminating the need to add them during a PCR step. The 5' phosphorylated reverse compliment of the 19 bp mosaic end (ME: 5'-Phos-CTG TCT CTT ATA CAC ATC T-3') sequence was hybridized to NoPCR1/2 by combining 5 μl of each NoPCR1 and NoPCR2 with 10 μl ME reverse complement all at 100 μM with 80 μl TE, followed by denaturation at 95°C for 5 min then slow cooling to room temperature for a final annealed adaptor concentration of 10 μM. Transposomes were assembled by incubating 5 μl annealed adaptors at 10 μM with 5 μl 100% glycerol and 10 μl Ez-Tn5 transposase (Epicentre) and allowed to incubate at room temperature for 20 min.

Tagmentation was carried out using previously prepared *E. coli *(CC118) or human (NA18507) genomic DNA using either 100 or 200 ng of DNA, 5 μl prepared transposomes, 2 μl 5× Nextera HMW buffer (Epicentre), and water to 10 μl. Reactions were incubated at 55°C for 5 min, followed by the addition of 25 μl 2× FailSafe PCR master mix (Epicentre), 1 μl FailSafe DNA polymerase (Epicentre), and 14 μl nuclease-free water (Ambion), and subsequent 5 min incubation at 72°C for nick translation. Tubes were then cleaned up using Qiaquick MinElute PCR purification columns (Qiagen), eluting in 12 μl buffer EB.

For each reaction, 2 μl was used as template for a real time PCR on a MiniOpticon (Bio-Rad) using 0.5 μl SYBR Green, 25 μl 2× Nextera PCR master mix, 1 μl Nextera PCR enzyme and nuclease-free water to 50 μl. Alongside the NoPCR reactions, libraries of known concentrations were used as template at successive dilutions to be used as a standard for rough library quantification. Standard Nextera cycling conditions were used, without the initial 72°C extension step. PCR reactions were cleaned up by Qiaquick PCR purification columns and run on a Noved 6% TBE PAGE gel (Invitrogen) for library size verification. After quantification, libraries were sequenced as per standard Illumina GAIIx protocol as a paired-end 36 bp run.

### Low input transposase-based library preparation

For the 500 pg and 100 pg *E. coli *(CC118) libraries and 10 pg human library (NA18507, Coriell), genomic DNA (in 1 μl volume) was incubated with 1 μl Nextera Illumina-compatible transposomes (Epicentre) at a 1 to 50 dilution (1 μl Nextera enzyme, 24 μl TE, 25 μl 100% glycerol), 1 μl 5× Nextera HMW buffer, and 2 μl nuclease-free water (Ambion). To avoid contamination, all dilutions and reaction preparation was carried out in a PCR hood. Reactions were incubated at 55°C for 5 min followed by addition of 25 μl 2× Nextera PCR buffer, 0.5 μl SYBR Green, 1 μl 50× Nextera primer cocktail, and 1 μl 0.5 μM barcode adaptor 2 (barcodes A6, A9, or A4 for 500 pg and 100 pg *E. coli *DNA, or 10 pg human DNA, respectively) and cycled under standard Nextera conditions in a MiniOpticon (Bio-Rad) real-time PCR thermocycler. Both reactions were removed after 20 cycles and cleaned up using Qiaquick MinElute columns, eluting in 20 μl EB. Libraries were run on a 6% Novex TBE PAGE gel (Invitrogen) for size verification and sequenced as barcoded spike-ins as per standard Illumina GAIIx protocol as a paired-end 101 bp (plus 9 bp barcode) run for *E. coli *libraries and a paired-end 36 bp (plus 9 bp barcode) run for human.

### Direct colony-based library preparation

Fusion-Blue chemically competent *E. coli *(Clontech, Mountain View, CA, USA) were transformed with pUC19 bearing a 2 kbp insert of human genomic DNA, and then plated on Luria broth (LB) + ampicillin. A small number of cells were picked from a single bacterial colony with a 10 μl pipette tip, and then transferred with dipping into 15 μl nuclease-free H_2_O. The suspended cells were heat-lysed at 95°C for 5 min, then placed on ice for 2 min. Nextera 5× LMW reaction buffer and enzyme (Illumina-compatible; Epicentre) were added to the sample, followed by brief mixing and incubation at 55°C for 5 min. The reaction was then stopped by heating to 70°C for 15 min. Sequencing-compatible primer sites were added in a 50 μl PCR reaction using 5 μl of the transposase reaction directly as template without intervening purification. PCR was carried out with 31.6 μl H_2_O, 10 μl Kapa 2G robust A buffer 5× (Kapa Biosystems, Cape Town, South Africa), 1 μl dNTP mix (10 mM each), 0.25 μl SYBR Green 100×, 1 μl 50× Nextera primer cocktail, 1 μl 50× Nextera adaptor 2, and 0.20 μl Kapa 2G robust polymerase; cycling conditions were as described by Epicentre. The amplification reaction was cleaned up with a Qiaquick PCR clean-up column (Qiagen) and eluted into 50 μl EB.

### Sequencing

Sequencing of the *H. sapiens *NA18507, *E. coli *CC118, and *D. melanogaster *libraries was done on an Illumina Genome Analyzer IIx as paired-end 36, 36, and 45 bp runs, respectively, using standard read primers for sonication and fragmentase libraries, run in individual lanes, and Nextera read primers for Nextera libraries. *H. sapiens *YH1 libraries were run on an Illumina HiSeq2000 as paired-end 90 bp run using Nextera read primers. Phage libraries contained library-specific barcodes and were run as multiplexed samples using GS FLX Titanium sequencing protocols. *P. aeruginosa *libraries were pooled by combining 100 ng of each strain library, and sequenced on an Illumina Genome Analyzer IIx with a paired-end 76-cycle run.

### Short read mapping

Short read mapping was done on the *E. coli *CC118, *D. melanogaster *and *H. sapiens *(NA18507 & YH1) Illumina GAIIx or HiSeq2000 sequenced samples by converting the raw sequence files to fastq format and then mapping to the hg18 (NCBI36) reference using the BWA [16] alignment software. After mapping, PCR duplicates were removed, as well as read-pairs with an insert size shorter than that of the read length.

### Long read assembly

Long read assembly from bacteriophage samples sequenced on the Roche GS FLX Ti was done using Roche's newbler assembler under default parameters. Individual reads from each dataset were mapped against the assembled genome using gsMapper (Roche Software Release: 2.3 (091027_1459)).

### Fragmentation site characterization

Fragmentation site characterization was carried out by stacking all regions of the genome flanking forward strand mapping start locations and the reverse complement of reverse strand start locations followed by calculating nucleotide frequencies at each position relative to the fragmentation site, thus generating a positional weight matrix (PWM). The PWMs then were imported into the SeqLogo (Oliver Bembom, Dept. of Biostatistics, University of California, Berkeley, 2008) package for Bioconductor in R and used to generate positional information content (IC) and sequence logos using the equation outlined by T. D. Schneider *et al. *[21,22]:

$$\text{IC(w)}={\text{log}}_{\text{2}}(\text{J})+\Sigma_{j=1}^{\text{J}}\;p_{wj}{\text{ log}}_{\text{2}}(p_{wj})={\text{log}}_{\text{2}}(\text{J})-entropy(w)$$

where J is the number of variables in the alphabet (4; A, C, G, or T), and *j *is the base at position *w*. This equation does not factor in the background nucleotide frequencies.

### Normalization of Illumina GAIIx coverage

Normalization of Illumina GAIIx coverage for the *E. coli *(non-size-selected) data was done by dividing the coverage at each position in the genome by the total number of mapped bases and then multiplying by a constant (the average number of mapped bases was close to 1 Gb, therefore 10^9 ^was used as the constant).

### Coverage distribution histograms

Coverage distribution histograms were generated by calculating the number of times each base of the genome was sequenced and plotting the frequency of each level of coverage.

### Coverage by G+C content

Coverage by G+C content plots were generated by binning the reference genome into 500 bp bins for *E. coli*, 10 kbp bins for human, and 1 kbp bins for *Drosophila *(other sizes were also investigated, resulting in very similar distributions) and calculating the G+C content of each bin, followed by plotting the coverage of that bin.

### Library complexity

Library complexity was calculated by random sampling of 50,000 read-pairs without replacement and plotting the number of uniquely occurring read-pairs versus the total number of sampled read-pairs.

### Insertion size distributions

Insertion size distributions were generated by taking the distance between the start mapping location of the first read and the end mapping location of the second read for every read-pair and plotting the frequency of occurrences of each insert size.

### SNP calls

SNP calls for the YH1 genome were generated using the SAMtools [18] variant caller with a maximum coverage of 1,000 and minimum quality score of 30. Prior to variant calling, read-pairs with an insert size less than 90 bp and reads not properly paired were removed to reduce noise. Calls were then compared with the SNPs reported by Wang *et al. *(2008) [15] and to dbSNP build 129.

### Cell-line-discordant SNP calls

In order to minimize false calls due to mapping errors, a repeat-masked version of hg18 was used along with further masking with respect to mappability according to the UCSC Genome Browser 'Rosetta 35mer uniqueness' and excluding all regions with a score of 0 (this score means that the sequence maps perfectly to multiple locations in the genome). This track was used because it was generated using the BWA aligner that was used in our analysis, and because the original YH1 sequence data is made up of 36 bp reads. Out of this newly masked genome, positions were called that had a SNP quality score in the cell line over 30, a reference call in blood over 30, and coverage less than 100× in both datasets. Of those with a quality score of 50 in both for their calls, 100 were randomly chosen for validation.

Validation was carried out using a mass spectrometry assay (Sequenom, San Diego, CA, USA). Primers for PCR amplification and extension were successfully designed for 100 mutation sites using the Sequenom MassArray Assay Design v3.1. PCR amplification, shrimp alkaline phosphatase treatment of unincorporated dNTPs, probe extension and resin desalting were carried out in sequence using the conditions described elsewhere [23]. Sequenom genotyping was performed in parallel for genomic DNA from YH1 blood and the same batch of lymphoblastoid cell lines as was used for sequencing. A negative control and technical replicate were also run in parallel for each typed position. Genotyping of all 100 testing sites passed the filter criteria of: (1) no failing extension, (2) no false positive in the negative control, (3) consistency between two technical replicates. Genotyping was further performed for the 100 positions in the YH primary lymphoblastoid cell lines using the same method, with 98 meeting the above filter criteria.

### Exome analysis

Sequence reads from transposase-based libraries subjected to human exome capture were aligned to the human reference (hg18) using BWA [16]. Each aligned base was deemed to be on target if it was within 100 bases of a targeted sequence. At each position within target regions, coverage was assessed and any position with a depth of at least one was considered covered. Comparison to standard exome methods was made by trimming read 1 of a PE76 lane from a GAIIx down to 36 bases and aligning it the same way. Because the standard library had fewer reads mapped, 28 million reads were randomly taken from both libraries and above analysis performed. Complexity was interrogated by taking an equivalent number of on-target SE36 reads generated by each method and calculating the percentage with unique start-points.

### Barcode design

Barcode design yielded a set of 96 × 9 bp sequences in which each 9 bp sequence contained no homopolymer run of three or more bases, had a GC content of ≤ 60%, was a edit distance of at least four away from all other members of the set of 96, and screened negative when compared with other adaptor and primer sequences used here. Also, we took care to ensure that each base (A, G, C, or T) was represented at least once in each 9 bp barcode, and at least once in each position along the 9 bp.

### Barcode deconvolution

Barcode deconvolution for pooled, multiplexed *Pseudomonas *samples was carried out by computing the Levenshtein edit distance between the obtained index read and each of the 96 barcode sequences used. The corresponding read-pair was assigned to a barcode when that barcode was within edit distance of 2 of the index read, with the next closest matching barcode being at least two further edits away.

### Data accessibility

All sequence data described here is being deposited in the NCBI Sequence Read Archive (SRA) under accession SRP004087.

## Competing interests

NCC is an employee of Epicentre Biotechnologies. The other authors declare that they have no completing interests.

## Authors' contributions

AA, HGM, Asan, XX, NCC, XZ, and JS conceived the study and planned experiments. AA analyzed all data, conducted comparison experiments with the Illumina platform, and developed the PCR-free protocol, the exome capture protocol, and the low-input protocol. HGM conducted comparison experiments and accompanying analyses with the 454 platform. Asan, XX, and XZ performed YH1 and *Drosophila *genome sequencing, and SNP validation. JOK developed the direct-from-colony library construction protocol and analyzed the resulting data. APM performed exome capture experiments. EHT, BS and AA developed and tested the 96-plex barcoding protocol. AA and JS wrote the manuscript. All authors reviewed drafts, contributed comments, and approved the final manuscript.

## Supplementary Material

Additional file 1 **Supplementary Tables 1-3**. Click here for file

Additional file 2 **Supplementary Figures 1-8**. Click here for file

Additional file 3 **Supplementary Table 4**. S4: barcode adaptors. Click here for file

## References

[B1] Kahvejian A Quackenbush J Thompson JF What would you do if you could sequence everything? Nat Biotechnol 2008 26 1125 1133 10.1038/nbt1494 18846086 PMC4153598

[B2] Shendure J Ji H Next-generation DNA sequencing. Nat Biotechnol 2008 26 1135 1145 10.1038/nbt1486 18846087

[B3] Bentley DR Balasubramanian S Swerdlow HP Smith GP Milton J Brown CG Hall KP Evers DJ Barnes CL Bignell HR Boutell JM Bryant J Carter RJ Keira Cheetham R Cox AJ Ellis DJ Flatbush MR Gormley NA Humphray SJ Irving LJ Karbelashvili MS Kirk SM Li H Liu X Maisinger KS Murray LJ Obradovic B Ost T Parkinson ML Pratt MR Accurate whole human genome sequencing using reversible terminator chemistry. Nature 2008 456 53 59 10.1038/nature07517 18987734 PMC2581791

[B4] Margulies M Egholm M Altman WE Attiya S Bader JS Bemben LA Berka J Braverman MS Chen YJ Chen Z Dewell SB Du L Fierro JM Gomes XV Godwin BC He W Helgesen S Ho CH Irzyk GP Jando SC Alenquer ML Jarvie TP Jirage KB Kim JB Knight JR Lanza JR Leamon JH Lefkowitz SM Lei M Li J Genome sequencing in microfabricated high-density picolitre reactors. Nature 2005 437 376 380 16056220 10.1038/nature03959 PMC1464427

[B5] McKernan KJ Peckham HE Costa GL McLaughlin SF Fu Y Tsung EF Clouser CR Duncan C Ichikawa JK Lee CC Zhang Z Ranade SS Dimalanta ET Hyland FC Sokolsky TD Zhang L Sheridan A Fu H Hendrickson CL Li B Kotler L Stuart JR Malek JA Manning JM Antipova AA Perez DS Moore MP Hayashibara KC Lyons MR Beaudoin RE Sequence and structural variation in a human genome uncovered by short-read, massively parallel ligation sequencing using two-base encoding. Genome Res 2009 19 1527 1541 10.1101/gr.091868.109 19546169 PMC2752135

[B6] Eid J Fehr A Gray J Luong K Lyle J Otto G Peluso P Rank D Baybayan P Bettman B Bibillo A Bjornson K Chaudhuri B Christians F Cicero R Clark S Dalal R Dewinter A Dixon J Foquet M Gaertner A Hardenbol P Heiner C Hester K Holden D Kearns G Kong X Kuse R Lacroix Y Lin S Real-time DNA sequencing from single polymerase molecules. Science 2009 323 133 138 10.1126/science.1162986 19023044

[B7] Mortazavi A Williams BA McCue K Schaeffer L Wold B Mapping and quantifying mammalian transcriptomes by RNA-Seq. Nat Methods 2008 5 621 628 10.1038/nmeth.1226 18516045 PMC13303166

[B8] Lennon NJ Lintner RE Anderson S Alvarez P Barry A Brockman W Daza R Erlich RL Giannoukos G Green L Hollinger A Hoover CA Jaffe DB Juhn F McCarthy D Perrin D Ponchner K Powers TL Rizzolo K Robbins D Ryan E Russ C Sparrow T Stalker J Steelman S Weiand M Zimmer A Henn MR Nusbaum C Nicol R A scalable, fully automated process for construction of sequence-ready barcoded libraries for 454. Genome Biol 11 R15 10.1186/gb-2010-11-2-r15 20137071 PMC2872875

[B9] Craig DW Pearson JV Szelinger S Sekar A Redman M Corneveaux JJ Pawlowski TL Laub T Nunn G Stephan DA Homer N Huentelman MJ Identification of genetic variants using bar-coded multiplexed sequencing. Nat Methods 2008 5 887 893 10.1038/nmeth.1251 18794863 PMC3171277

[B10] Quail MA Kozarewa I Smith F Scally A Stephens PJ Durbin R Swerdlow H Turner DJ A large genome center's improvements to the Illumina sequencing system. Nat Methods 2008 5 1005 1010 10.1038/nmeth.1270 19034268 PMC2610436

[B11] Kozarewa I Ning Z Quail MA Sanders MJ Berriman M Turner DJ Amplification-free Illumina sequencing-library preparation facilitates improved mapping and assembly of (G+C)-biased genomes. Nat Methods 2009 6 291 295 10.1038/nmeth.1311 19287394 PMC2664327

[B12] Mamanova L Andrews RM James KD Sheridan EM Ellis PD Langford CF Ost TW Collins JE Turner DJ FRT-seq: amplification-free, strand-specific transcriptome sequencing. Nat Methods 2010 7 130 132 10.1038/nmeth.1417 20081834 PMC2861772

[B13] Reznikoff WS Tn5 as a model for understanding DNA transposition. Mol Microbiol 2003 47 1199 1206 10.1046/j.1365-2958.2003.03382.x 12603728

[B14] Reznikoff WS Transposon Tn5. Annu Rev Genet 2008 42 269 286 10.1146/annurev.genet.42.110807.091656 18680433

[B15] Wang J Wang W Li R Li Y Tian G Goodman L Fan W Zhang J Li J Guo Y Feng B Li H Lu Y Fang X Liang H Du Z Li D Zhao Y Hu Y Yang Z Zheng H Hellmann I Inouye M Pool J Yi X Zhao J Duan J Zhou Y Qin J Ma L The diploid genome sequence of an Asian individual. Nature 2008 456 60 65 10.1038/nature07484 18987735 PMC2716080

[B16] Li H Durbin R Fast and accurate short read alignment with Burrows-Wheeler transform. Bioinformatics 2009 25 1754 1760 10.1093/bioinformatics/btp324 19451168 PMC2705234

[B17] Goryshin IY Miller JA Kil YV Lanzov VA Reznikoff WS Tn5/IS50 target recognition. Proc Natl Acad Sci USA 1998 95 10716 10721 10.1073/pnas.95.18.10716 9724770 PMC27961

[B18] Li H Handsaker B Wysoker A Fennell T Ruan J Homer N Marth G Abecasis G Durbin R The Sequence Alignment/Map format and SAMtools. Bioinformatics 2009 25 2078 2079 10.1093/bioinformatics/btp352 19505943 PMC2723002

[B19] Manoil C Beckwith J TnphoA: a transposon probe for protein export signals. Proc Natl Acad Sci USA 1985 82 8129 8133 10.1073/pnas.82.23.8129 2999794 PMC391456

[B20] Blin N Stafford DW A general method for isolation of high molecular weight DNA from eukaryotes. Nucleic Acids Res 1976 3 2303 2308 987581 10.1093/nar/3.9.2303 PMC343085

[B21] Schneider TD Stormo GD Gold L Ehrenfeucht A Information content of binding sites on nucleotide sequences. J Mol Biol 1986 188 415 431 10.1016/0022-2836(86)90165-8 3525846

[B22] Schneider TD Stephens RM Sequence logos: a new way to display consensus sequences. Nucleic Acids Res 1990 18 6097 6100 10.1093/nar/18.20.6097 2172928 PMC332411

[B23] Yi X Liang Y Huerta-Sanchez E Jin X Cuo ZX Pool JE Xu X Jiang H Vinckenbosch N Korneliussen TS Zheng H Liu T He W Li K Luo R Nie X Wu H Zhao M Cao H Zou J Shan Y Li S Yang Q Asan Ni P Tian G Xu J Liu X Jiang T Wu R Sequencing of 50 human exomes reveals adaptation to high altitude. Science 329 75 78 10.1126/science.1190371 20595611 PMC3711608

