# Supplementary material for: Rapid, low-input, low-bias construction of shotgun fragment libraries by high-density in vitro transposition
Source: Genome Biol. 2010 Dec 8;11(12):R119. doi: 10.1186/gb-2010-11-12-r119 (PMC3046479; doi:10.1186/gb-2010-11-12-r119)
Supplement: Additional file 1 — Supplementary Tables 1-3. [file gb-2010-11-12-r119-S1.pdf]

Supplementary Table 1

Summary of libraries sequenced in comparative analysis

Organism	Method	Size Selection	Sequencing Platform	Raw Read Pairs	Mapper	Raw Mapped	PCR Duplicates Removed†	Unique Mapped %	Complexity
	Sonication, replicate 1	none	Illumina GAIIx (PE36)	14,764,330	BWA	13,096,300	12,719,017	86%	97%
	Sonication, replicate 2	none	Illumina GAIIx (PE36)	15,177,716	BWA	13,283,051	13,018,271	86%	98%
	Endonuclease, replicate 1	none	Illumina GAIIx (PE36)	15,131,182	BWA	13,374,850	12,775,619	84%	96%
	Endonuclease, replicate 2	none	Illumina GAIIx (PE36)	16,171,788	BWA	14,124,278	13,441,857	83%	95%
	Transposase, replicate 1	none	Illumina GAIIx (PE36)	19,812,714	BWA	16,439,998	13,209,901	67%	80%
	Transposase, replicate 2	none	Illumina GAIIx (PE36)	23,239,433	BWA	17,501,733	14,094,636	61%	81%
	Transposase	none	Illumina GAIIx (PE76)	47,445,883	BWA	32,763,991	24,138,660	51%	74%
**E. coli CC118**	Transposase	Ampure >300	Illumina GAIIx (PE76)	49,202,822	BWA	28,370,120	21,618,428	44%	76%
	Transposase	Caliper 350+/-10%	Illumina GAIIx (PE76)	14,781,578	BWA	12,898,508	9,439,353	64%	73%
	Transposase NoPCR (100ng)	none	Illumina GAIIx (PE36)	N/A	BWA	512,202	510,620	N/A	100%
	Transposase NoPCR (200ng)	none	Illumina GAIIx (PE36)	N/A	BWA	1,492,999	1,475,918	N/A	99%
	Transposase NoPCR (50ng), 1	none	Illumina GAIIx (PE101)	spike in	BWA	417,268	414,738	N/A	99%
	Transposase NoPCR (50ng), 2	none	Illumina GAIIx (PE101)	spike in	BWA	416,216	413,342	N/A	99%
	Transposase (500pg)	none	Illumina GAIIx (PE101)	spike in	BWA	882,236	814,763	N/A	92%
	Transposase (100pg)	none	Illumina GAIIx (PE101)	spike in	BWA	1,232,098	1,097,646	N/A	89%
	Sonication, replicate 1**	none	Illumina GAIIx (PE36)	8,830,236	BWA	8,515,999	8,451,436	96%	99%
	Sonication, replicate 2**	none	Illumina GAIIx (PE36)	8,779,733	BWA	8,471,772	8,403,393	96%	99%
	Endonuclease, replicate 1	none	Illumina GAIIx (PE36)	10,137,423	BWA	8,499,831	8,295,592	82%	98%
	Endonuclease, replicate 2	none	Illumina GAIIx (PE36)	12,477,053	BWA	10,790,123	10,637,932	85%	99%
**H. sapiens NA18507**	Transposase, replicate 1	none	Illumina GAIIx (PE36)	18,378,252	BWA	15,814,133	15,602,480	85%	99%
	Transposase, replicate 2	none	Illumina GAIIx (PE36)	18,559,340	BWA	15,877,850	15,653,955	84%	99%
	Transposase NoPCR (100ng)	none	Illumina GAIIx (PE36)	N/A	BWA	1,044,539	1,037,373	N/A	99%
	Transposase NoPCR (200ng)	none	Illumina GAIIx (PE36)	N/A	BWA	488,026	485,966	N/A	99%
	Transposase (10pg)††	none	Illumina GAIIx (PE36)	N/A	BWA	5,731,004	2,182,087	N/A	38%
	Nebulization	none	Roche GS FLX Ti*	46,745	gsMapper	43,707	43,707	94%	N/A
**CRW10**	Endonuclease	none	Roche GS FLX Ti*	6,444	gsMapper	5,983	5,983	93%	N/A
	Transposase	none	Roche GS FLX Ti*	47,986	gsMapper	43,917	43,917	92%	N/A
	Nebulization	none	Roche GS FLX Ti*	6,183	gsMapper	6,121	6,121	>99%	N/A
**PA1**	Endonuclease	none	Roche GS FLX Ti*	9,979	gsMapper	9,412	9,412	94%	N/A
	Transposase	none	Roche GS FLX Ti*	43,639	gsMapper	40,637	40,637	93%	N/A
**D. melanogaster w1118**	Transposase (2rxn)	Gel 400-450	Illumina GAIIx (PE45)	31,272,321	BWA	30,918,821	28,142,311	90%	91%
	Transposase (5rxn)	Gel 550-575	Illumina HiSeq2000 (PE90)	103,341,810	BWA	82,511,867	81,183,426	79%	98%
	Transposase (2rxn)	Gel 400-500	Illumina HiSeq2000 (PE90)	68,022,215	BWA	62,183,263	60,709,520	89%	98%
		Gel 400-500	Illumina HiSeq2000 (PE90)	67,823,613	BWA	62,293,546	60,817,189	90%	98%
**H. sapiens YH1**		Gel 500-550	Illumina HiSeq2000 (PE90)	58,597,591	BWA	51,335,787	49,590,370	85%	97%
		Gel 550-650	Illumina HiSeq2000 (PE90)	52,625,788	BWA	47,144,748	45,258,959	86%	96%
	Transposase (1rxn)	Gel 300-500	Illumina HiSeq2000 (PE90)	68,342,929	BWA	58,105,589	56,850,508	83%	98%
		Gel 500-650	Illumina HiSeq2000 (PE90)	54,039,263	BWA	44,013,398	42,200,046	78%	96%
**H. sapiens BK229.03**	Transposase – Exome Capture	none	Illumina GAIIx (SE36)*	44,472,457	BWA	34,654,705	N/A	78%	N/A

* Single end read only

** Data for sonication method for human libraries taken as single lanes of data from Bentley et al. Nature 2008

† PCR Duplicates removed using the samtools rmdup function.

†† Duplicates removed by using outer mapping coordinates.

Supplementary Table 2

Information content of sequence bias in vicinity of fragmentation sites

Information Content (-10 to +15bp)

		Average	Maximum
**E. coli CC118**	**Sonication**	0.007	0.102
	**Endonuclease**	0.015	0.108
	**Transposase**	0.046	0.157
**H. sapiens NA18507**	**Sonication**	0.031	0.048
	**Endonuclease**	0.024	0.144
	**Transposase**	0.049	0.153
**CRW10**	**Nebulization**	0.049	0.063
	**Endonuclease**	0.050	0.134
	**Transposase**	0.052	0.109
**PA1**	**Nebulization**	0.018	0.035
	**Endonuclease**	0.032	0.085
	**Transposase**	0.026	0.053

Supplementary Table 3

Pearson Correlation of Coverage (upper triangle); Spearman Rank Order Correlation of Coverage (lower triangle)

	Son.1	Son.2	End.1	End.2	Tr.1	Tr.2
**Son.1**		0.816	0.653	0.678	0.336	0.366
**Son.2**	0.854		0.688	0.691	0.320	0.370
**End.1**	0.693	0.696		0.795	0.383	0.376
**End.2**	0.720	0.720	0.818		0.352	0.401
**Tr.1**	0.409	0.421	0.435	0.428		0.835
**Tr.2**	0.489	0.497	0.490	0.493	0.877	
