# Supplementary material for: Rapid, low-input, low-bias construction of shotgun fragment libraries by high-density in vitro transposition
Source: Genome Biol. 2010 Dec 8;11(12):R119. doi: 10.1186/gb-2010-11-12-r119 (PMC3046479; doi:10.1186/gb-2010-11-12-r119)
Supplement: Additional file 2 — Supplementary Figures 1-8. [file gb-2010-11-12-r119-S2.pdf]

Supplementary Figure 1

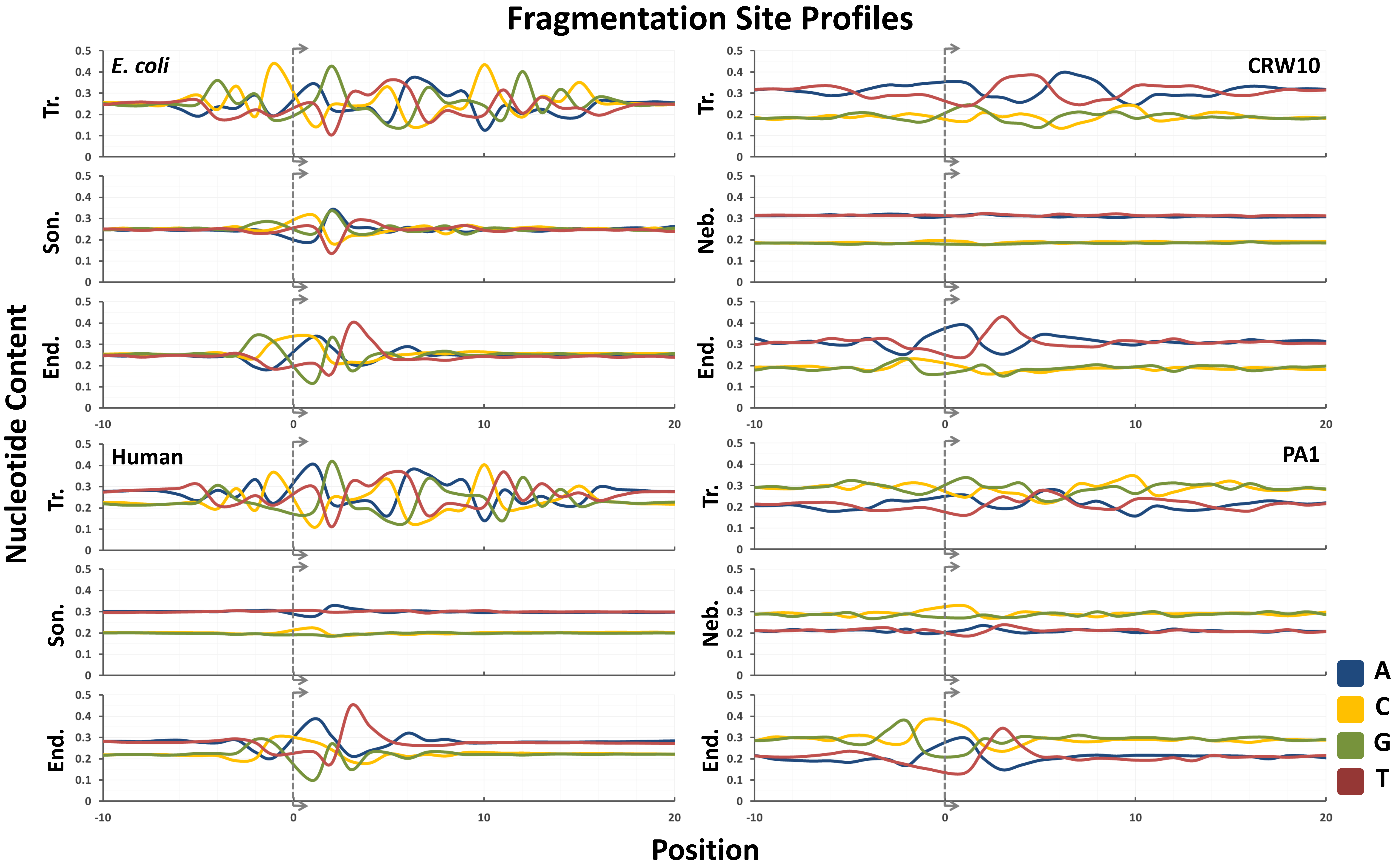

Fragmentation site profiles. Nucleotide content over a 30 bp interval (-10, +20) corresponding to read starts for each method of library construction (Tr. = Transposase, Son. = Sonication, Neb. = Nebulization, End. = Endonuclease). Observed biases are shown for Illumina GAIIx sequenced libraries (*E. coli* (CC118), human (NA18507)) and Roche (454) GSFLX sequenced phage libraries (CRW10, PA1).

Supplementary Figure 2

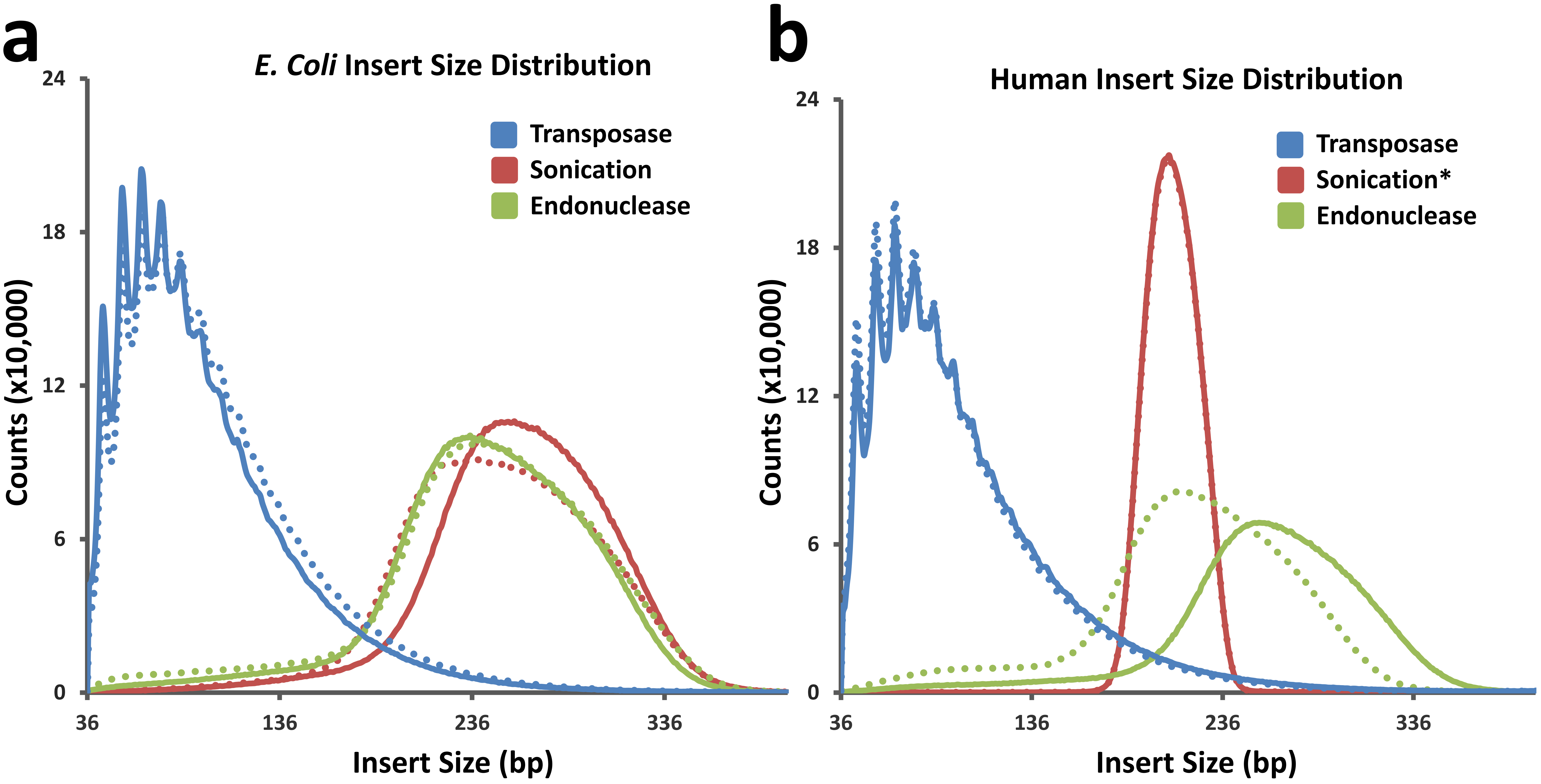

Insert size distribution for *E. coli* CC118 **(a)** and *H. sapiens* NA18507 **(b)** libraries of each method sequenced on the Illumina GAIIx platform with replicates (dashed). *The *H. sapiens* library prepared by sonication is from Bentley *et al.* and had two size selections resulting in a tight distribution. In both organisms, the transposase based method shows a slight periodicity at ~10 bp intervals, likely due to physical constraints on the ability for a transposase to attack certain positions with regards to the helical pitch of the DNA as it extends away from the bound transposase.

Supplementary Figure 3

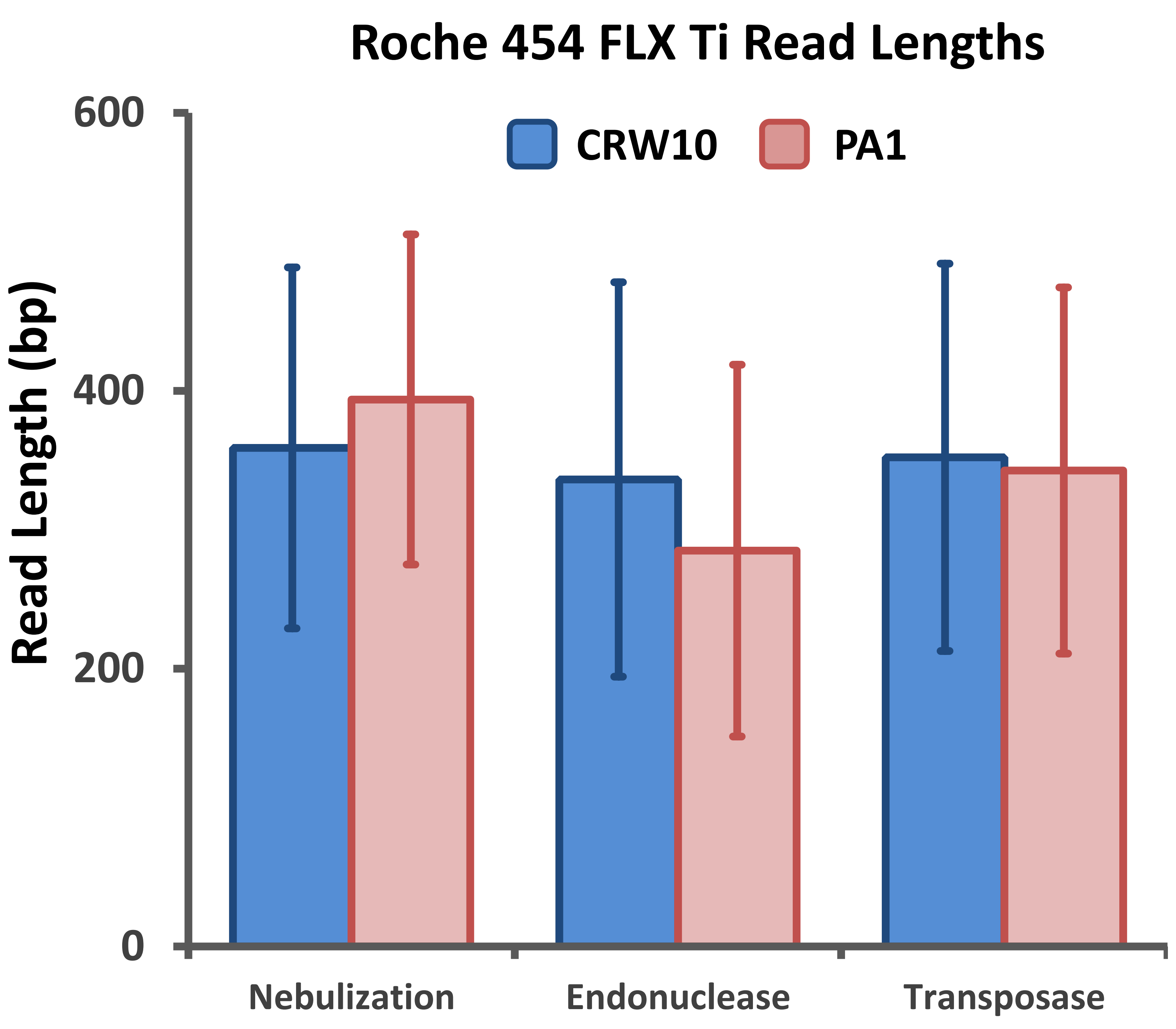

Average read lengths for Roche (454) GS FLX Ti libraries for CRW10 and PA1 bacteriophages using nebulization, endonuclease, and transposase methods.

Supplementary Figure 4

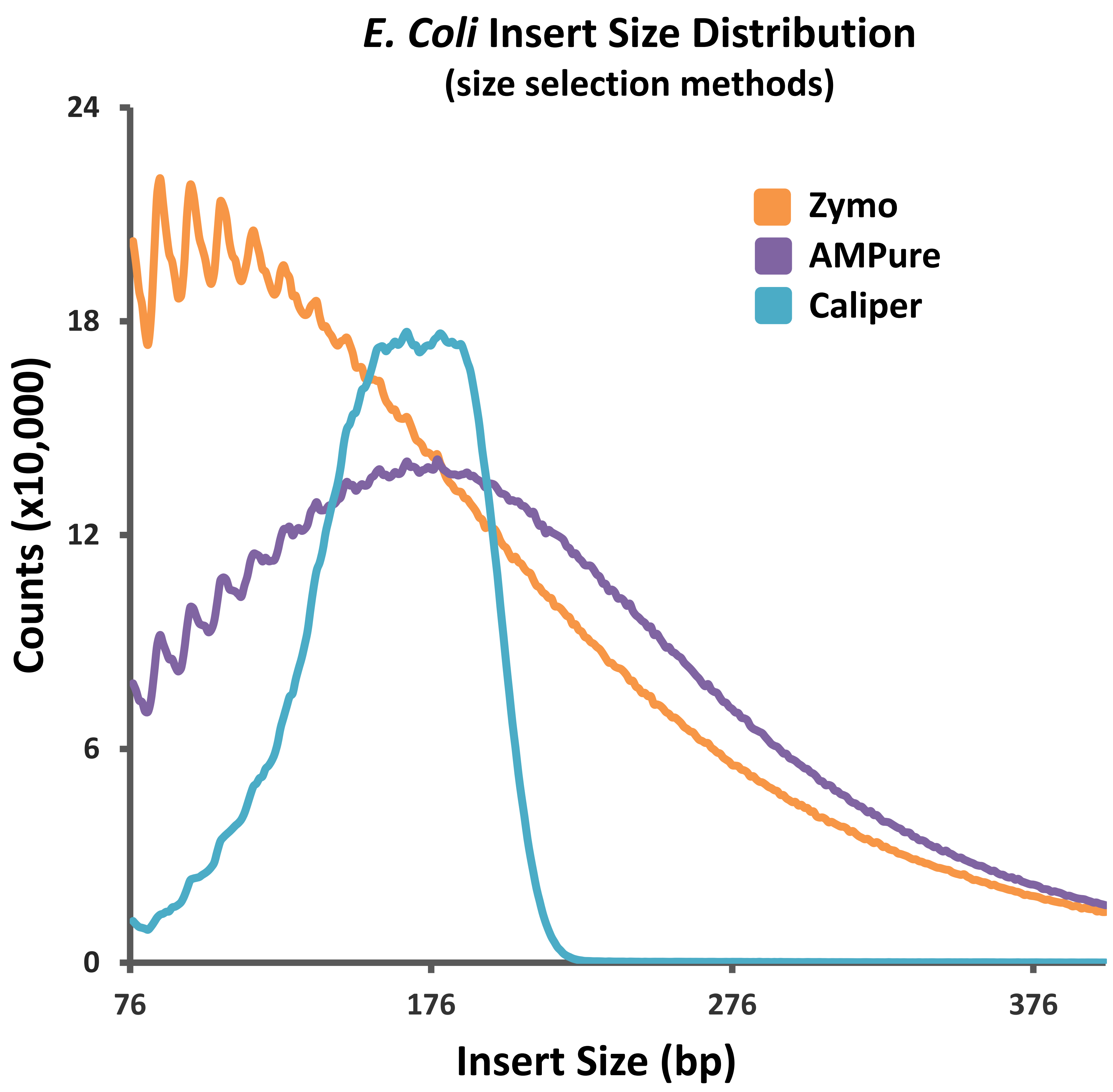

Insert size distribution for *E. coli* CC118 after three different size selection methods: Zymo column clean-up (orange), AMPure bead clean-up (300 bp cutoff, purple), and Caliper chip-based size selection (350+/-10%bp, teal).

Supplementary Figure 5

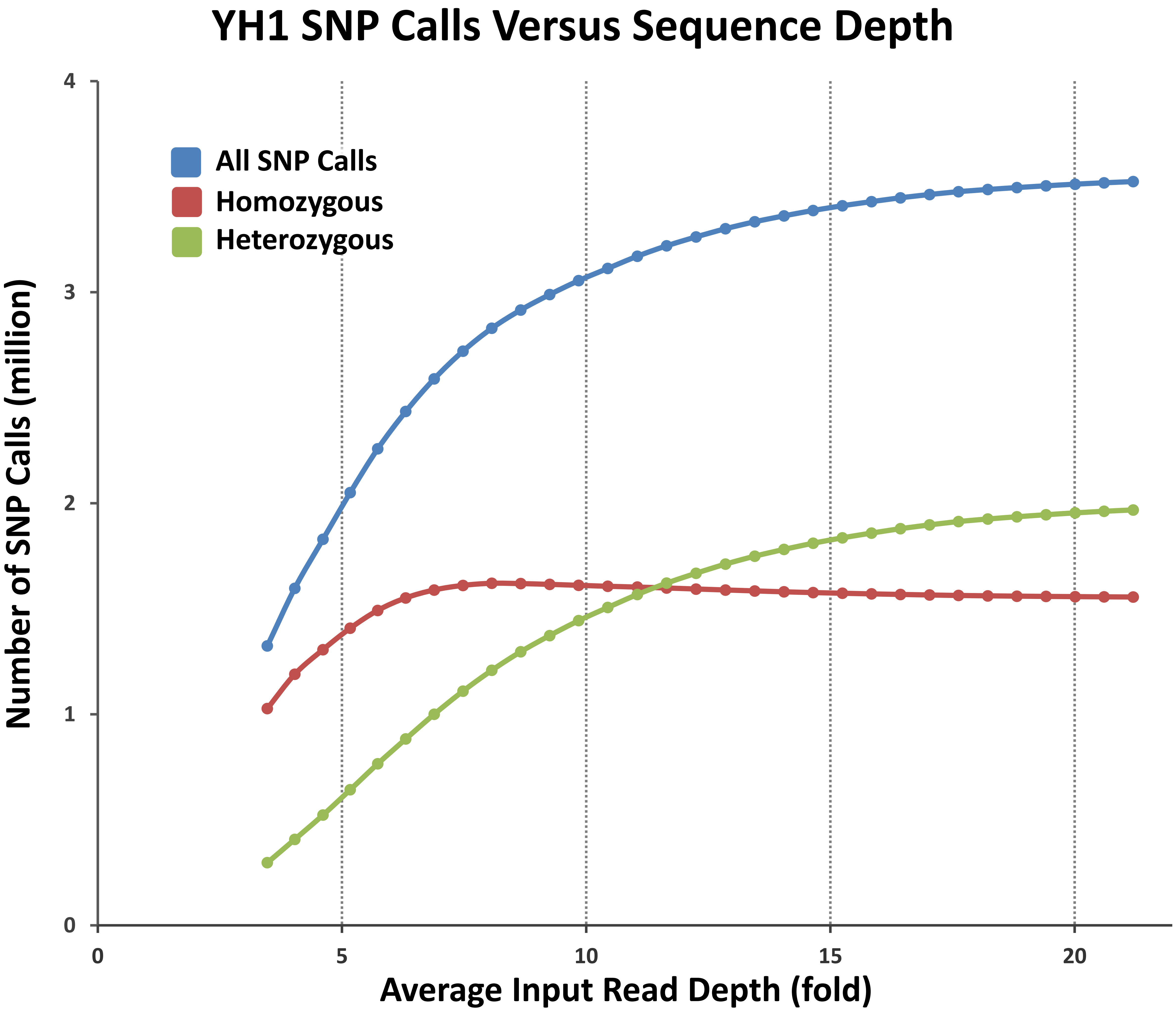

SNP calls versus sequence depth were calculated by pulling random sets of 10 million mapped read pairs (properly paired, insert size >=90 bp) without replacement and calling SNP positions of a minimum quality of 30. Trend observed is consistent with previously reported whole genome analyses (Bentley *et. al.* (2008)).

Supplementary Figure 6

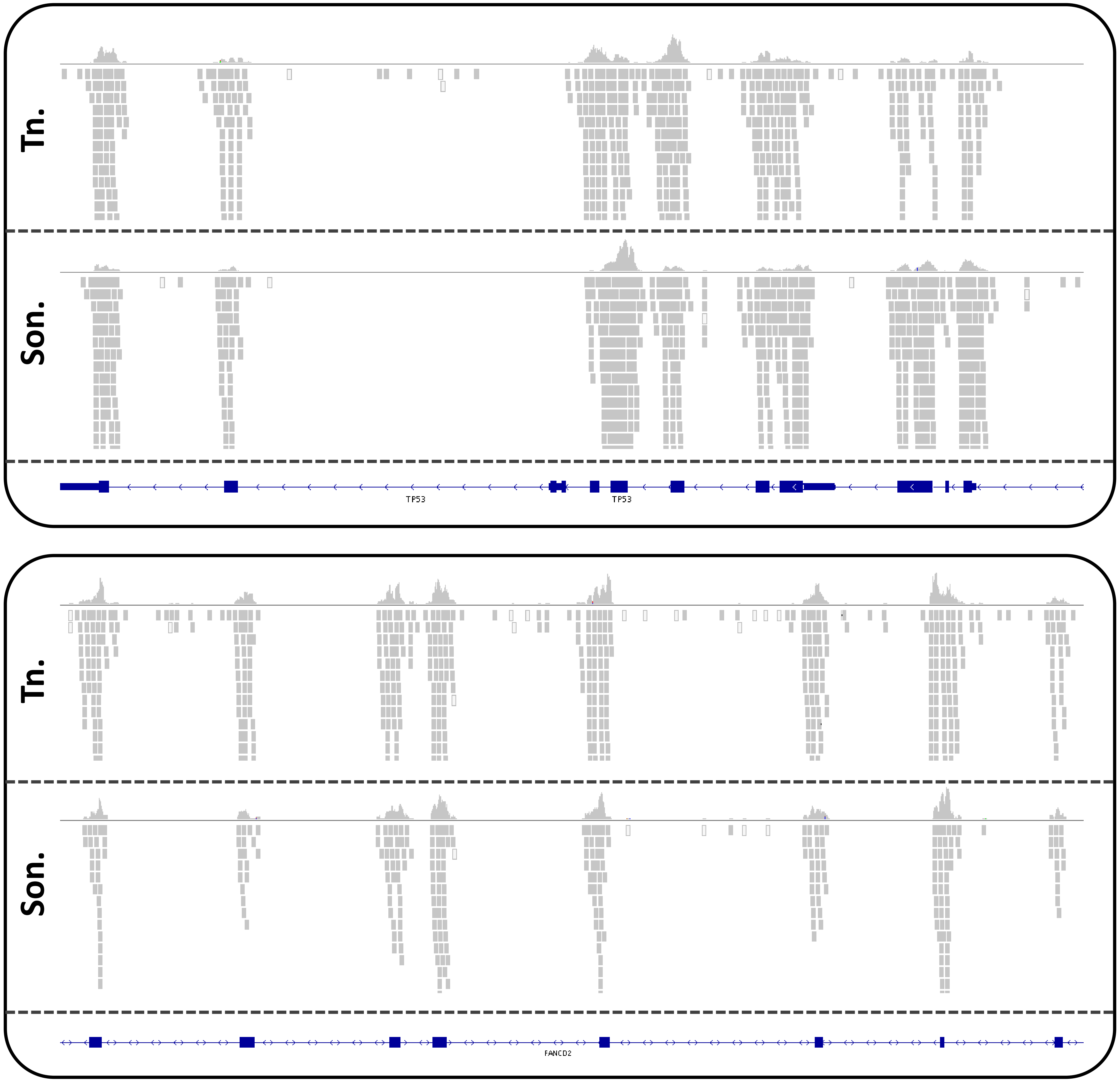

Sample views of exome sequencing comparison. Standard (sonication-based) libraries from 3ug of starting material and transposase-based libraries from 50ng of starting material were subjected to whole human exome target capture and sequenced on an Illumina GAIIx. Shown are two typical example views of reads aligned to the genome for an identical number of mapped reads of each method. (Tn. = Transposase-based, Son. = Sonication-based)

Supplementary Figure 7

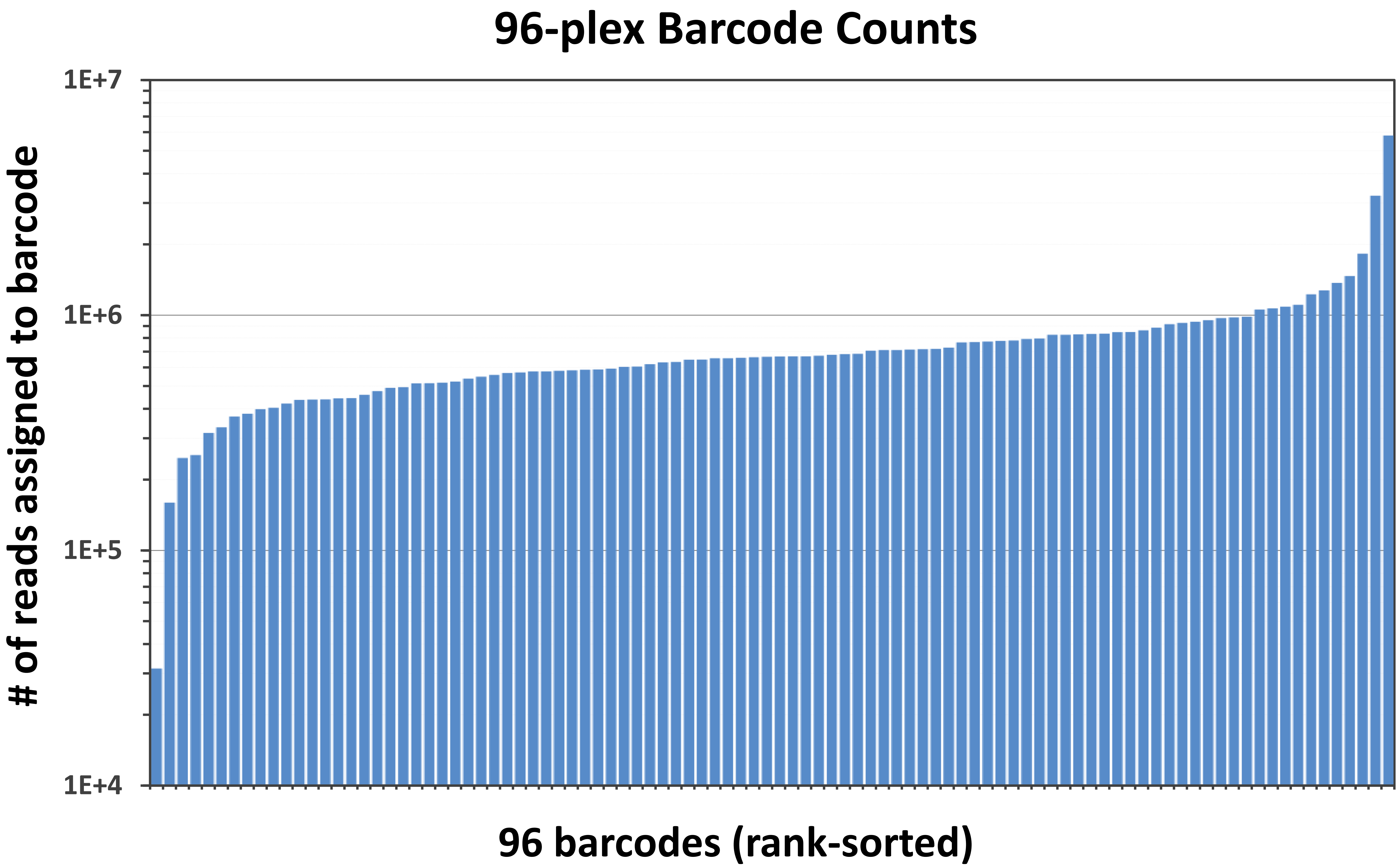

Performance of sample indexing. Libraries were constructed from 96 DNAs using adaptors bearing 9 bp index sequences. Samples were quantified by Nanodrop prior to normalized pooling. The number of reads assigned to each index is plotted here. 90% (86 of 96) fall within a 4-fold range of relative abundance.

Supplementary Figure 8

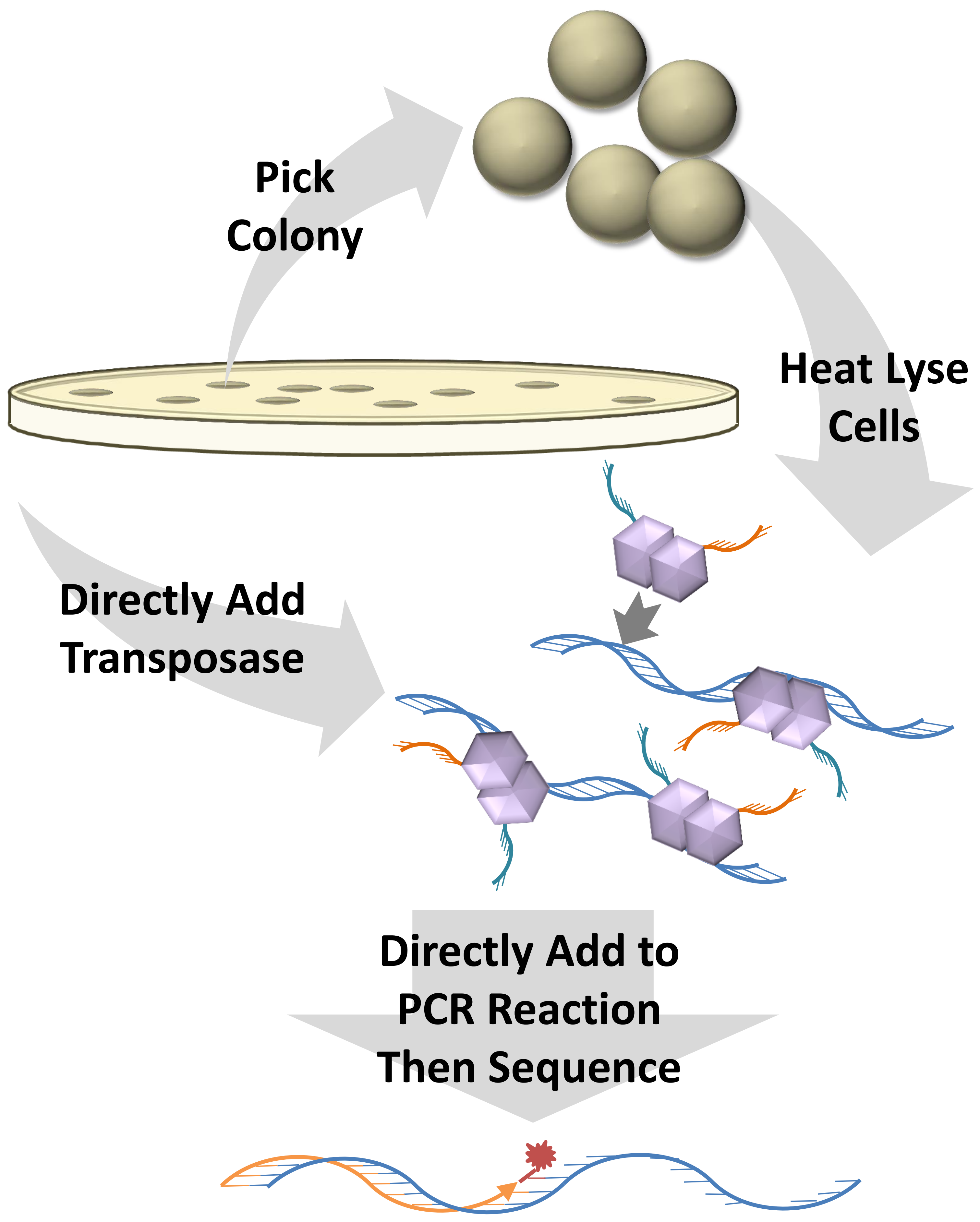

“Colony PCR” based approach to library preparation diagram detailing the simplicity of the method. A colony is picked using a pipette tip followed by lysing the cells by boiling. The lysate is then used as the material for transposase-catalyzed adaptor insertion without clean-up then directly used in the PCR reaction, also without cleanup. After PCR a column purification is performed and the library is sequencer ready.
