# Supplementary material for: Rapid, low-input, low-bias construction of shotgun fragment libraries by high-density in vitro transposition
Source: Genome Biol. 2010 Dec 8;11(12):R119. doi: 10.1186/gb-2010-11-12-r119 (PMC3046479; doi:10.1186/gb-2010-11-12-r119)
Supplement: Additional file 3 — Supplementary Table 4. S4: barcode adaptors. [file gb-2010-11-12-r119-S3.pdf]

Supplementary Table 4. Primers and barcodes for 96-plex sample indexing

Standard "Adaptor 2"

CAAGCAGAAGACGGCATACGAGATCGGTCTGCCTTGCCAGCCCGCTCAG

Format for modified "Adaptor 2"

CAAGCAGAAGACGGCATACGAGAT#########CGGTCTGCCTTGCCAGCCCGCTCAG

Adaptor 2 with embedded barcode (9 bp x 96 sequences)	Cell # for IDT plate	Barcodes alone
CAAGCAGAAGACGGCATACGAGATTACGAAGTCCGGTCTGCCTTGCCAGCCCGCTCAG	A1	TACGAAGTC
CAAGCAGAAGACGGCATACGAGATGACGAGATTCGGTCTGCCTTGCCAGCCCGCTCAG	B1	GACGAGATT
CAAGCAGAAGACGGCATACGAGATACCGTAAGACGGTCTGCCTTGCCAGCCCGCTCAG	C1	ACCGTAAGA
CAAGCAGAAGACGGCATACGAGATTAGTGGCAACGGTCTGCCTTGCCAGCCCGCTCAG	D1	TAGTGGCAA
CAAGCAGAAGACGGCATACGAGATCATTAACGCCGGTCTGCCTTGCCAGCCCGCTCAG	E1	CATTAACGC
CAAGCAGAAGACGGCATACGAGATTCGTTGAAGCGGTCTGCCTTGCCAGCCCGCTCAG	F1	TCGTTGAAG
CAAGCAGAAGACGGCATACGAGATTAGTACGCTCGGTCTGCCTTGCCAGCCCGCTCAG	G1	TAGTACGCT
CAAGCAGAAGACGGCATACGAGATCTCAGATCACGGTCTGCCTTGCCAGCCCGCTCAG	H1	CTCAGATCA
CAAGCAGAAGACGGCATACGAGATTTCACCGTACGGTCTGCCTTGCCAGCCCGCTCAG	A2	TTCACCGTA
CAAGCAGAAGACGGCATACGAGATGTCATGCATCGGTCTGCCTTGCCAGCCCGCTCAG	B2	GTCATGCAT
CAAGCAGAAGACGGCATACGAGATAGGACAGTTCGGTCTGCCTTGCCAGCCCGCTCAG	C2	AGGACAGTT
CAAGCAGAAGACGGCATACGAGATATGGTGTCTCGGTCTGCCTTGCCAGCCCGCTCAG	D2	ATGGTGTCT
CAAGCAGAAGACGGCATACGAGATGGATGTTCTCGGTCTGCCTTGCCAGCCCGCTCAG	E2	GGATGTTCT
CAAGCAGAAGACGGCATACGAGATCTTATCCAGCGGTCTGCCTTGCCAGCCCGCTCAG	F2	CTTATCCAG
CAAGCAGAAGACGGCATACGAGATGTAAGTCACCGGTCTGCCTTGCCAGCCCGCTCAG	G2	GTAAGTCAC
CAAGCAGAAGACGGCATACGAGATTTCAGTGAGCGGTCTGCCTTGCCAGCCCGCTCAG	H2	TTCAGTGAG
CAAGCAGAAGACGGCATACGAGATCTCGTAATGCGGTCTGCCTTGCCAGCCCGCTCAG	A3	CTCGTAATG
CAAGCAGAAGACGGCATACGAGATCATGTCTCACGGTCTGCCTTGCCAGCCCGCTCAG	B3	CATGTCTCA
CAAGCAGAAGACGGCATACGAGATAATCGTGGACGGTCTGCCTTGCCAGCCCGCTCAG	C3	AATCGTGGA
CAAGCAGAAGACGGCATACGAGATGTATCAGTCCGGTCTGCCTTGCCAGCCCGCTCAG	D3	GTATCAGTC
CAAGCAGAAGACGGCATACGAGATAGCAGATGTCGGTCTGCCTTGCCAGCCCGCTCAG	E3	AGCAGATGT
CAAGCAGAAGACGGCATACGAGATTCCTAACGTCGGTCTGCCTTGCCAGCCCGCTCAG	F3	TCCTAACGT
CAAGCAGAAGACGGCATACGAGATAACAGTCCACGGTCTGCCTTGCCAGCCCGCTCAG	G3	AACAGTCCA
CAAGCAGAAGACGGCATACGAGATCCTTGAGAACGGTCTGCCTTGCCAGCCCGCTCAG	H3	CCTTGAGAA
CAAGCAGAAGACGGCATACGAGATTTAAGCCTGCGGTCTGCCTTGCCAGCCCGCTCAG	A4	TTAAGCCTG
CAAGCAGAAGACGGCATACGAGATTTAGACCACCGGTCTGCCTTGCCAGCCCGCTCAG	B4	TTAGACCAC
CAAGCAGAAGACGGCATACGAGATTGTCTAGTGCGGTCTGCCTTGCCAGCCCGCTCAG	C4	TGTCTAGTG
CAAGCAGAAGACGGCATACGAGATTAGATCGAGCGGTCTGCCTTGCCAGCCCGCTCAG	D4	TAGATCGAG
CAAGCAGAAGACGGCATACGAGATTGAATGCCACGGTCTGCCTTGCCAGCCCGCTCAG	E4	TGAATGCCA
CAAGCAGAAGACGGCATACGAGATGTGCAATGTCGGTCTGCCTTGCCAGCCCGCTCAG	F4	GTGCAATGT
CAAGCAGAAGACGGCATACGAGATAGTGGCATACGGTCTGCCTTGCCAGCCCGCTCAG	G4	AGTGGCATA
CAAGCAGAAGACGGCATACGAGATATGATCGGTCGGTCTGCCTTGCCAGCCCGCTCAG	H4	ATGATCGGT
CAAGCAGAAGACGGCATACGAGATAGTCTACCTCGGTCTGCCTTGCCAGCCCGCTCAG	A5	AGTCTACCT
CAAGCAGAAGACGGCATACGAGATGATCAACTGCGGTCTGCCTTGCCAGCCCGCTCAG	B5	GATCAACTG
CAAGCAGAAGACGGCATACGAGATATCGGTAGTCGGTCTGCCTTGCCAGCCCGCTCAG	C5	ATCGGTAGT
CAAGCAGAAGACGGCATACGAGATCGTATGATGCGGTCTGCCTTGCCAGCCCGCTCAG	D5	CGTATGATG
CAAGCAGAAGACGGCATACGAGATTTACTGACGCGGTCTGCCTTGCCAGCCCGCTCAG	E5	TTACTGACG
CAAGCAGAAGACGGCATACGAGATCTGTCGTAACGGTCTGCCTTGCCAGCCCGCTCAG	F5	CTGTCGTAA
CAAGCAGAAGACGGCATACGAGATTCAACTGGTCGGTCTGCCTTGCCAGCCCGCTCAG	G5	TCAACTGGT
CAAGCAGAAGACGGCATACGAGATATCGATCTCCGGTCTGCCTTGCCAGCCCGCTCAG	H5	ATCGATCTC
CAAGCAGAAGACGGCATACGAGATGCAACTATGCGGTCTGCCTTGCCAGCCCGCTCAG	A6	GCAACTATG
CAAGCAGAAGACGGCATACGAGATGATGACTTCCGGTCTGCCTTGCCAGCCCGCTCAG	B6	GATGACTTC
CAAGCAGAAGACGGCATACGAGATGACGTTACACGGTCTGCCTTGCCAGCCCGCTCAG	C6	GACGTTACA

CAAGCAGAAGACGGCATACGAGATCATCTGCTACGGTCTGCCTTGCCAGCCCGCTCAG	D6	CATCTGCTA
CAAGCAGAAGACGGCATACGAGATATTAGTCGGCGGTCTGCCTTGCCAGCCCGCTCAG	E6	ATTAGTCGG
CAAGCAGAAGACGGCATACGAGATTAGCGTACTCGGTCTGCCTTGCCAGCCCGCTCAG	F6	TAGCGTACT
CAAGCAGAAGACGGCATACGAGATCCAAGCAATCGGTCTGCCTTGCCAGCCCGCTCAG	G6	CCAAGCAAT
CAAGCAGAAGACGGCATACGAGATCCGTAATTGCGGTCTGCCTTGCCAGCCCGCTCAG	H6	CCGTAATTG
CAAGCAGAAGACGGCATACGAGATAGAATTGCCCGGTCTGCCTTGCCAGCCCGCTCAG	A7	AGAATTGCC
CAAGCAGAAGACGGCATACGAGATACCTGTAACCGGTCTGCCTTGCCAGCCCGCTCAG	B7	ACCTGTAAC
CAAGCAGAAGACGGCATACGAGATCATCAGTGTCGGTCTGCCTTGCCAGCCCGCTCAG	C7	CATCAGTGT
CAAGCAGAAGACGGCATACGAGATGAATCCTCACGGTCTGCCTTGCCAGCCCGCTCAG	D7	GAATCCTCA
CAAGCAGAAGACGGCATACGAGATGCTGTATACCGGTCTGCCTTGCCAGCCCGCTCAG	E7	GCTGTATAC
CAAGCAGAAGACGGCATACGAGATGAAGGCTATCGGTCTGCCTTGCCAGCCCGCTCAG	F7	GAAGGCTAT
CAAGCAGAAGACGGCATACGAGATGGAATCGATCGGTCTGCCTTGCCAGCCCGCTCAG	G7	GGAATCGAT
CAAGCAGAAGACGGCATACGAGATGCTTATGGTCGGTCTGCCTTGCCAGCCCGCTCAG	H7	GCTTATGGT
CAAGCAGAAGACGGCATACGAGATTGACGCATTCGGTCTGCCTTGCCAGCCCGCTCAG	A8	TGACGCATT
CAAGCAGAAGACGGCATACGAGATCACGATTCTCGGTCTGCCTTGCCAGCCCGCTCAG	B8	CACGATTCT
CAAGCAGAAGACGGCATACGAGATTATTGCCTCCGGTCTGCCTTGCCAGCCCGCTCAG	C8	TATTGCCTC
CAAGCAGAAGACGGCATACGAGATAAGTCAGAGCGGTCTGCCTTGCCAGCCCGCTCAG	D8	AAGTCAGAG
CAAGCAGAAGACGGCATACGAGATATAGCTGAGCGGTCTGCCTTGCCAGCCCGCTCAG	E8	ATAGCTGAG
CAAGCAGAAGACGGCATACGAGATTGCTCACAACGGTCTGCCTTGCCAGCCCGCTCAG	F8	TGCTCACAA
CAAGCAGAAGACGGCATACGAGATGTCTTCTGACGGTCTGCCTTGCCAGCCCGCTCAG	G8	GTCTTCTGA
CAAGCAGAAGACGGCATACGAGATTTGCCGATTCGGTCTGCCTTGCCAGCCCGCTCAG	H8	TTGCCGATT
CAAGCAGAAGACGGCATACGAGATCTCGAATACCGGTCTGCCTTGCCAGCCCGCTCAG	A9	CTCGAATAC
CAAGCAGAAGACGGCATACGAGATTGGCTTCTACGGTCTGCCTTGCCAGCCCGCTCAG	B9	TGGCTTCTA
CAAGCAGAAGACGGCATACGAGATAAGGCCATTCGGTCTGCCTTGCCAGCCCGCTCAG	C9	AAGGCCATT
CAAGCAGAAGACGGCATACGAGATAAGTTGACCCGGTCTGCCTTGCCAGCCCGCTCAG	D9	AAGTTGACC
CAAGCAGAAGACGGCATACGAGATCTGAACTGACGGTCTGCCTTGCCAGCCCGCTCAG	E9	CTGAACTGA
CAAGCAGAAGACGGCATACGAGATCTAGGTGTACGGTCTGCCTTGCCAGCCCGCTCAG	F9	CTAGGTGTA
CAAGCAGAAGACGGCATACGAGATCCATCTTAGCGGTCTGCCTTGCCAGCCCGCTCAG	G9	CCATCTTAG
CAAGCAGAAGACGGCATACGAGATCTACGACATCGGTCTGCCTTGCCAGCCCGCTCAG	H9	CTACGACAT
CAAGCAGAAGACGGCATACGAGATTCCAACATGCGGTCTGCCTTGCCAGCCCGCTCAG	A10	TCCAACATG
CAAGCAGAAGACGGCATACGAGATGCTATCATCCGGTCTGCCTTGCCAGCCCGCTCAG	B10	GCTATCATC
CAAGCAGAAGACGGCATACGAGATACAGCTTCACGGTCTGCCTTGCCAGCCCGCTCAG	C10	ACAGCTTCA
CAAGCAGAAGACGGCATACGAGATAGTCATTGCCGGTCTGCCTTGCCAGCCCGCTCAG	D10	AGTCATTGC
CAAGCAGAAGACGGCATACGAGATAGATCTCGACGGTCTGCCTTGCCAGCCCGCTCAG	E10	AGATCTCGA
CAAGCAGAAGACGGCATACGAGATATGCTCTTGCGGTCTGCCTTGCCAGCCCGCTCAG	F10	ATGCTCTTG
CAAGCAGAAGACGGCATACGAGATTTAGTGCGTCGGTCTGCCTTGCCAGCCCGCTCAG	G10	TTAGTGCGT
CAAGCAGAAGACGGCATACGAGATTCCTAGTTCCGGTCTGCCTTGCCAGCCCGCTCAG	H10	TCCTAGTTC
CAAGCAGAAGACGGCATACGAGATGGTGCATTACGGTCTGCCTTGCCAGCCCGCTCAG	A11	GGTGCATTA
CAAGCAGAAGACGGCATACGAGATACTGAGGATCGGTCTGCCTTGCCAGCCCGCTCAG	B11	ACTGAGGAT
CAAGCAGAAGACGGCATACGAGATTAGCAGTCACGGTCTGCCTTGCCAGCCCGCTCAG	C11	TAGCAGTCA
CAAGCAGAAGACGGCATACGAGATTCACTCGAACGGTCTGCCTTGCCAGCCCGCTCAG	D11	TCACTCGAA
CAAGCAGAAGACGGCATACGAGATACCAATCAGCGGTCTGCCTTGCCAGCCCGCTCAG	E11	ACCAATCAG
CAAGCAGAAGACGGCATACGAGATGATATGGACCGGTCTGCCTTGCCAGCCCGCTCAG	F11	GATATGGAC
CAAGCAGAAGACGGCATACGAGATTGAGAGATCCGGTCTGCCTTGCCAGCCCGCTCAG	G11	TGAGAGATC
CAAGCAGAAGACGGCATACGAGATTGCCATTAGCGGTCTGCCTTGCCAGCCCGCTCAG	H11	TGCCATTAG
CAAGCAGAAGACGGCATACGAGATACTAACGCACGGTCTGCCTTGCCAGCCCGCTCAG	A12	ACTAACGCA
CAAGCAGAAGACGGCATACGAGATATGTAGCACCGGTCTGCCTTGCCAGCCCGCTCAG	B12	ATGTAGCAC
CAAGCAGAAGACGGCATACGAGATGGTCGATATCGGTCTGCCTTGCCAGCCCGCTCAG	C12	GGTCGATAT
CAAGCAGAAGACGGCATACGAGATGCGAGTTATCGGTCTGCCTTGCCAGCCCGCTCAG	D12	GCGAGTTAT
CAAGCAGAAGACGGCATACGAGATGACTGAGTACGGTCTGCCTTGCCAGCCCGCTCAG	E12	GACTGAGTA
CAAGCAGAAGACGGCATACGAGATAGATACTCCCGGTCTGCCTTGCCAGCCCGCTCAG	F12	AGATACTCC
CAAGCAGAAGACGGCATACGAGATGCTAGAGTTCGGTCTGCCTTGCCAGCCCGCTCAG	G12	GCTAGAGTT
CAAGCAGAAGACGGCATACGAGATAATGTAGCGCGGTCTGCCTTGCCAGCCCGCTCAG	H12	AATGTAGCG
